# Systematic discovery of mutation-specific synthetic lethals by mining pan-cancer human primary tumor data

**DOI:** 10.1038/ncomms15580

**Published:** 2017-05-31

**Authors:** Subarna Sinha, Daniel Thomas, Steven Chan, Yang Gao, Diede Brunen, Damoun Torabi, Andreas Reinisch, David Hernandez, Andy Chan, Erinn B. Rankin, Rene Bernards, Ravindra Majeti, David L. Dill

**Affiliations:** 1 Department of Computer Science, Stanford University, Stanford, California 94305, USA; 2 Department of Medicine, Division of Hematology, Cancer Institute, and Institute for Stem Cell Biology and Regenerative Medicine, Stanford University School of Medicine, Stanford, California 94305, USA; 3 Princess Margaret Cancer Centre, University Health Network, Toronto, Ontario, Canada M5G 2M9; 4 Department of Electrical Engineering and Computer Science, University of California at Berkeley, Berkeley, California 94720, USA; 5 Division of Molecular Carcinogenesis, The Netherlands Cancer Institute, Plesmanlaan 121, Amsterdam 1066 CX, The Netherlands; 6 Division of Radiation and Cancer Biology, Department of Radiation Oncology, Stanford University School of Medicine, Stanford, California 94305, USA; 7 Department of Obstetrics and Gynecology, Stanford University School of Medicine, Stanford, California 94305, USA

## Abstract

Two genes are synthetically lethal (SL) when defects in both are lethal to a cell but a single defect is non-lethal. SL partners of cancer mutations are of great interest as pharmacological targets; however, identifying them by cell line-based methods is challenging. Here we develop MiSL (Mining Synthetic Lethals), an algorithm that mines pan-cancer human primary tumour data to identify mutation-specific SL partners for specific cancers. We apply MiSL to 12 different cancers and predict 145,891 SL partners for 3,120 mutations, including known mutation-specific SL partners. Comparisons with functional screens show that MiSL predictions are enriched for SLs in multiple cancers. We extensively validate a SL interaction identified by MiSL between the *IDH1* mutation and *ACACA* in leukaemia using gene targeting and patient-derived xenografts. Furthermore, we apply MiSL to pinpoint genetic biomarkers for drug sensitivity. These results demonstrate that MiSL can accelerate precision oncology by identifying mutation-specific targets and biomarkers.

## Abstract (web summary)

There are no robust methods for systematically identifying mutation-specific synthetic lethal (SL) partners in cancer. Here, the authors develop a computational algorithm that uses pan-cancer data to detect mutation-andcancer-specific SL partners and they validate a novel SL interaction between mutant IDH and loss of ACACA in leukaemia.

It is now common to sequence for somatic mutations in patient tumours before treatment, but the identification of mutation-specific therapies remains a pivotal challenge for precision medicine. A particularly promising approach is to identify alternative therapies that do not target the mutation directly. For instance, a mutation may increase dependence on a second gene that can be easily targeted instead. In this case, the mutation and the second gene are called a synthetic lethal (SL) pair, since a defect in either gene is compatible with cell viability, but defects in both are lethal to the cell^1^,^2^, and the second gene is a SL partner of the original mutation.

Large-scale functional screens in cell lines using short hairpin RNA (shRNA)^3^,^4^,^5^,^6^,^7^, CRISPR^8^ or small-molecule libraries^9^ are widely used for high-throughput identification of SL interactions. While being a valuable technique to identify novel SL interactions, these functional screens suffer from limitations. Since experimental screens are usually performed in cell lines, they can be negatively impacted by: (1) the limited representation of newly discovered mutations in existing cell lines: for example, the Cancer Cell Line Encyclopedia (CCLE) collection of 1,000 cell lines contains no acute myeloid leukaemia (AML) cell line with an oncogenic *IDH1* mutation, even though the mutation is present in 10% of AML patients^10^, and (2) the artificiality of *in vitro* screening conditions^11^,^12^, which cannot fully capture *in vivo* tumour evolution in the tumour microenvironment. Furthermore, such screens may be limited by factors such as false positive hits due to off-target effects^13^ and false negatives due to limited coverage and incomplete gene knockdown in shRNA screens, and false positives due to genomic instability in CRISPR screens^14^,^15^.

New computational methods based on human primary tumour data are needed to identify mutation-specific SL partners to complement the limitations of existing cell line screening methods. Current computational methods to detect SL interactions use human orthologues of yeast SL interactions^16^,^17^, protein–protein networks^18^ or metabolic network analysis^19^,^20^. These approaches rely on incomplete data and/or network models and data that are not fully representative of human primary tumours. A recent computational method, called DAISY^21^, used tumour genomic data and shRNA data from cell lines to predict SL interactions. DAISY predicted a global network of potential SL interactions in human cells and marks an important advance in computational methods for predicting SL interactions in cancer. However, DAISY primarily utilizes a small number of inactivating (nonsense and frameshift) mutations and uses shRNA data from existing cell lines as part of its inference strategy, which means it will miss SL interactions that are false negatives in shRNA screens caused either by incomplete genetic knockdown or by inadequate representation of mutations in existing cell lines.

To address these limitations, we have developed MiSL (Mining Synthetic Lethals), a novel algorithm based on Boolean implications mined from large pan-cancer patient data sets to identify SL partners for specific cancer mutations in specific cancer types. We validate MiSL by (1) showing concordance between our predictions and mutation-specific SL partners identified by existing Achilles screens and our own functional screen, (2) finding known SL partners in AML and kidney cancer, and (3) demonstrating same pathway enrichment of the predicted SL partners, which is consistent with previous work in yeast. We also demonstrate that MiSL solves two problems that are directly translatable to clinical applications: identifying novel mutation-specific SL interactions, in particular *IDH1* mutation and *ACACA* in AML, and pinpointing predictive genetic biomarkers that can guide precise targeting of existing therapies.

## Results

### The MiSL algorithm

**Figure 1:**
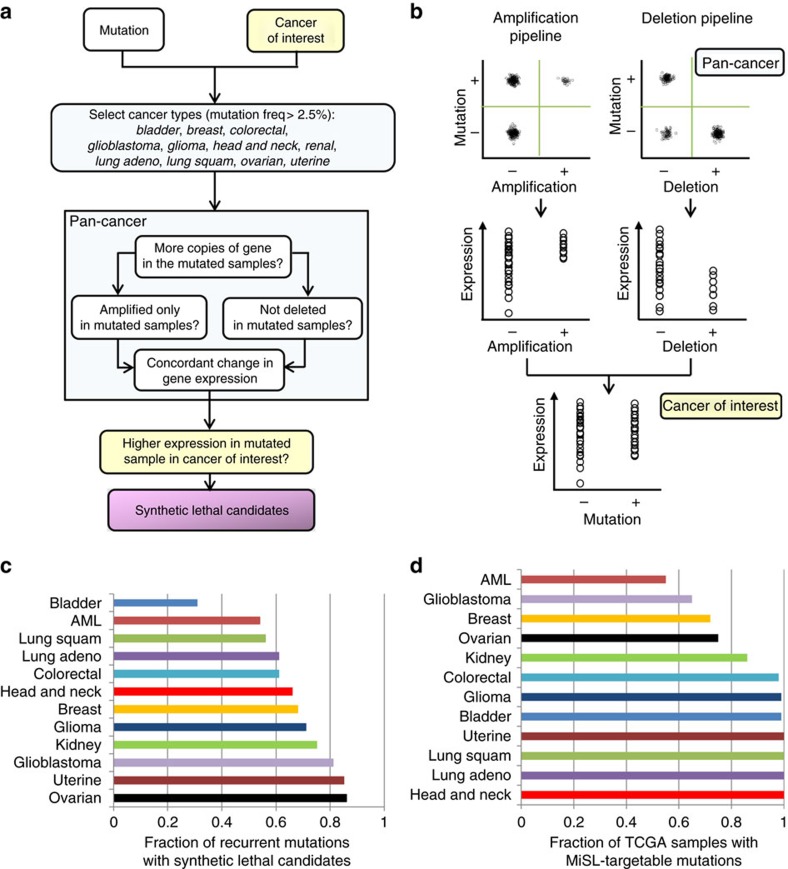
The MiSL algorithm. (**a**) Pipeline of MiSL algorithm: For a given mutation and a cancer of interest, the pipeline outputs a list of genes, which are the candidate SL partners or MiSL candidates of the mutation in the cancer of interest. (**b**) Depiction of the various steps through the deletion pipeline and amplification pipeline in MiSL. (**c**) Fraction of recurrent mutations with SL candidates in each of the 12 TCGA cancers. (**d**) Fraction of TCGA samples with MiSL-targetable mutations in each of the 12 TCGA cancers.

MiSL is a computational pipeline to identify candidate SL partners of mutations for subsequent focussed experimental validation using high-throughput pan-cancer primary tumour data sets (Fig. 1a). The starting point is a mutation and a cancer type of interest. For the results here, we utilized 12 cancer data sets from The Cancer Genome Atlas (TCGA)^22^. Our underlying assumption is that, across multiple cancers, SL partners of a mutation will be amplified more frequently or deleted less frequently in primary tumour samples harbouring the mutation, with concordant changes in expression. The output of MiSL is a relatively short, high-quality list of candidate SLs that must then be validated experimentally to find the true SLs. The reported results are based on data from approximately 3,000 primary tumour samples that are used to identify candidate SL partners of each recurrent mutation in each of the 12 cancer types (Supplementary Fig. 1a).

**Figure 2:**
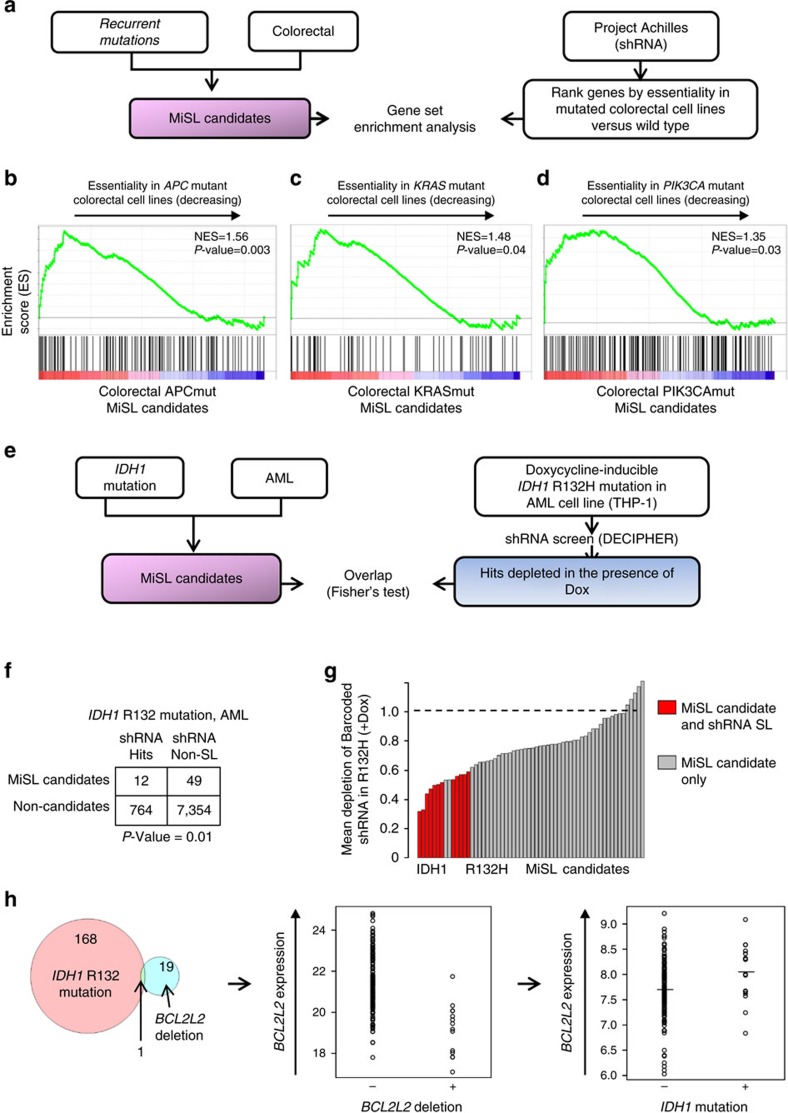
MiSL predictions are enriched for mutation-specific SL partners. (**a**) Schematic for comparing MiSL predictions for mutations in colorectal cancer with essential genes as per the shRNA data from Project Achilles. (**b**–**d**) Gene set enrichment plots assessing the degree of enrichment of MiSL candidates for mutations in colorectal cancer using the data from Project Achilles. Genes were ranked according to selective essentiality in mutated colorectal cancer cell lines versus wild-type cells using the shRNA data from Project Achilles. Enrichment analysis was done using GSEA to compare the ranked list of genes for each mutation with its MiSL candidates in colorectal cancer. A positive enrichment score meant that MiSL candidates were ranked near the top of the list for each mutation. (**b**) Enrichment plot for *APC* mutation in colorectal cancer (NES=1.55, *P* value=0.003). (**c**) Enrichment plot for *KRAS* mutation in colorectal cancer (NES=1.48, *P* value=0.04). (**d**) Enrichment plot for *PIK3CA* mutation in colorectal cancer (NES=1.35, *P* value=0.03). (**e**) Schematic for comparing MiSL candidates for *IDH1* mutation in AML with SL partners as per DECIPHER library screen generated using a Dox-inducible IDH1 R132H THP-1 cell line. (**f**) Overlap between MiSL candidates and SL partners as per the DECIPHER screen for the *IDH1* mutation in AML is statistically significant (*P* value=0.01, Fisher's exact test). (**g**) Plot summarizing average shRNA scores for hairpins for the 61 MiSL candidates represented in the DECIPHER screen. (**h**) MiSL analysis steps illustrated for *BCL2L2*—(i) mutual exclusion of *IDH1* mutation and gene deletion across cancers (HI-LO Boolean implication), (ii) deletion of gene concordant with lower expression of gene (*P*<0.05) and (iii) expression of gene is higher in *IDH1*-mutated AML (*P*<0.05).

Given a mutation *X* and a cancer type of interest, the analysis focusses on cancers in which *X* is present in at least 2.5% of the samples. Next, genes are identified that have more copies in the presence of a mutation as determined by: (1) preferred amplification in the presence of the mutation (amplification pipeline; Fig. 1b), or (2) deletion only in the absence of the mutation (deletion pipeline; Fig. 1b). Boolean implications^23^,^24^ (statistical IF–THEN relationships) are used to efficiently extract the required relationships from genomic data. For the amplification pipeline, we search for cases where *B* is amplified only in the presence of mutation *X* and thereby capture cases where there is dependence on gene *B* in the presence of mutation *X*. In other words, the logical statement ‘if gene *B* is amplified, then mutation *X* is present' holds for almost all samples. This relationship is called a HI-HI Boolean implication (Fig. 1b, Supplementary Fig. 1b). Similarly, for the deletion pipeline, we search for cases where almost all the samples where *B* is deleted are mutually exclusive with samples that have mutation *X* and thereby capture cases where co-occurrence of deletions in gene *B* and mutation *X* reflect a loss of fitness and hence are under-represented in the tumour population. In other words, the logical statement ‘if mutation *X* is present, then gene *B* is NOT deleted' holds for almost all samples. This is a HI-LO Boolean implication with mutation *X* (Fig. 1b, Supplementary Fig. 1c). To eliminate candidate genes that are passengers in large chromosomal alterations, we require that gene *B* is associated with concordant differential expression in samples that have the copy number alteration (CNA) versus those that do not in the included tumour types. Finally, the resulting gene set is filtered to include only those genes that are differentially overexpressed in the presence of the mutation versus the wild type in the cancer of interest. This step eliminates genes that are unlikely to be essential in the context of the cancer of interest and the specific mutation and also eliminates some false positives due to convergent evolution. Genes that satisfy all the above-mentioned filters form the set of candidate SL partners for a given mutation in a given cancer and will henceforth be called the MiSL candidates of the mutation. Furthermore, a mutation with a non-zero number of MiSL candidates will be called MiSL-targetable.

MiSL identified candidate SL partners for 3,120 recurrent mutations, which spans a large fraction (0.3–0.8) of recurrent mutations in each of the 12 cancers (Fig. 1c). For the majority of recurrent mutations in each cancer type, MiSL identified <50 candidate SL partners (Supplementary Fig. 1d, MiSL candidates for each mutation and cancer type in Supplementary Data 1), providing a focussed list of candidates for experimental testing. Additionally, the MiSL candidates were robust to changes in the *P* value thresholds of the various filters, since the majority of candidates were retained even when the *P* value thresholds were halved (Supplementary Fig. 1e). Many MiSL-targetable mutations had common MiSL candidates across different cancer types (Supplementary Data 2). Out of the 1,084 mutations with MiSL candidates in >1 cancer, 60% had common MiSL candidates across multiple cancer types (Supplementary Fig. 1f), suggesting that some mutation-specific SL partners are applicable across different cancers. Finally, the majority of samples had MiSL-targetable mutations, demonstrating the potential of the method to find new treatment options for large numbers of patients (Fig. 1d).

### MiSL predictions are enriched for mutation-specific SL partners

To assess the quality of predictions from MiSL, we compared its candidates with SL partners identified using shRNA screens in cell lines as these remain the most prevalent high-throughput approach used to find SLs. This comparison does not assume that shRNA screens find only true SL partners, only that their results are probably better than random. Thus we would expect to see some concordance between MiSL candidates and the results of such screens. We compared MiSL results with shRNA knockdown data for approximately 11,000 genes in 216 cell lines from Project Achilles^25^. We focussed on colorectal cancer because it had the most evaluable mutations (Supplementary Fig. 2a). A mutation was considered evaluable if it was MiSL-targetable and present in more than five cell lines in that cancer type, and there was sufficient overlap between MiSL candidates for the mutation and genes for which shRNA data were available. For the evaluable mutations, which included *APC*, *CSMD3*, *KRAS*, *PIK3CA* and *TP53* (Supplementary Fig. 2a), we compared MiSL predictions with the results from Achilles (Fig. 2a). First, we performed a differential analysis of the scores for each shRNA in mutated colorectal cancer cell lines versus wild-type lines. A summary score was generated for each gene by combining the differential analysis results of all shRNAs targeting the gene. The genes were subsequently ranked using these scores with more essential genes near the top of the list (more details in Supplementary Methods). For the majority of the evaluable mutations in colorectal cancer (Supplementary Fig. 2b), enrichment analysis using gene set enrichment analysis (GSEA)^26^ revealed that MiSL candidates were preferentially ranked higher by mutation-specific essentiality in colorectal cancer (Fig. 2b–d, Supplementary Fig. 2c–e) and included statistically significant results for *APC* (normalized enrichment score (NES)=1.55, *P* value=0.003), *KRAS* (NES=1.48, *P* value=0.04) and *PIK3CA* (NES=1.35, *P* value=0.03). This indicates that MiSL indeed identifies mutation-specific SL partners in specific cancer types. However, many of the candidates identified by MiSL were not present in Achilles shRNA libraries, indicating that MiSL identifies SL partners that large-scale screens miss.

Next, we sought to systematically investigate the MiSL candidates for the *IDH1* mutation in AML. We focussed on the *IDH1* mutation since we had previously developed an inducible AML cell line that expresses mutant IDH1 (ref. 27), providing us with an appropriate experimental system to test MiSL candidates. We performed an independent shRNA library screen (DECIPHER) for the *IDH1* R132 mutation expressed in THP-1 cells using a doxycycline (Dox)-inducible promoter^27^ and compared the shRNA predictions with MiSL candidates (Fig. 2e). MiSL predicted 89 candidate SL partners for the *IDH1* mutation in AML (Supplementary Data 1, details of pipeline steps in Supplementary Data 3). We used the DECIPHER library for the shRNA screen, which covered 61 out of the 89 candidates. A gene was considered to be an SL partner according to the shRNA screen, if at least two shRNA hairpins targeting the gene produced a >20% reduction in barcoded reads in the presence of mutant IDH1 (+Dox) versus the control (−Dox). Comparing the gene lists of shRNA SL partners and the MiSL candidates for the *IDH1* mutation in AML produced a statistically significant overlap (*P* value=0.01, Fisher's exact test, Fig. 2f), indicating that MiSL candidates were enriched for genes whose knockdown resulted in preferential cell death. Using a more stringent criterion for determining shRNA SL partners (>40% reduction in viability per hairpin) also produced a statistically significant overlap with MiSL candidates (*P* value=0.004, Fisher's exact test, Supplementary Fig. 2f). Finally, for a majority of MiSL candidates, the average scores of the two shRNA hairpins with the most dropout in the presence of the mutation had a value <1.0 (Fig. 2g), consistent with some degree of mutation-specific synthetic lethality.

Most interestingly, MiSL identified *BCL2L2* (Bcl-w) as a candidate SL partner of the *IDH1* mutation: *IDH1* mutation and *BCL2L2* deletion were mutually exclusive (HI-LO Boolean implication) in the TCGA data; *BCL2L2* deletion resulted in lowered expression, so *BCL2L2* is unlikely to be a passenger deletion; and *BCL2L2* was differentially overexpressed in *IDH1*-mutant compared to *IDH1*-wild-type AML (Fig. 2h). This is noteworthy since a SL interaction between Bcl-2 family members and *IDH1* mutation in AML was recently identified and validated using *in vitro* and xenograft models^27^, constituting an example of a true mutation-specific SL partner emerging from the deletion pipeline in MiSL (Fig. 1b). Similarly, the MiSL amplification pipeline uncovered a previously known SL interaction: *GLS* as a SL partner of the *VHL* mutation in kidney cancer. *GLS* was selectively amplified only in the presence of the *VHL* mutation (HI-HI Boolean implication), *GLS* amplification resulted in increased expression of *GLS*, and *GLS* was differentially overexpressed in *VHL*-mutant compared to *VHL*-wild-type kidney cancer (Supplementary Fig. 2g). This prediction is consistent with previous work that showed a selective *in vivo* dependence on the glutaminase pathway for VHL mutants in kidney cancer^28^. Additionally, our own experimental data using *VHL*-deficient and isogenic *VHL*-restored RCC4 cell lines confirmed that targeting *GLS* with siRNAs showed a significant reduction in cell viability (Supplementary Fig. 2h). These data indicate that mutual exclusion relationships with gene deletions, as well as subset relationships with gene amplifications arise due to synthetic lethality. Furthermore, the concordance between MiSL predictions and shRNA data for different combinations of mutations and cancers confirms that MiSL output is enriched for mutation-specific SL partners in a specific tumour type.

We would also expect MiSL candidates to be enriched for the same pathways as the mutation, since previous reports in yeast have indicated that SL interactions often occur between genes belonging to the same pathway or process^29^. SL interactions in human cells are also known to occur between genes in the same biological process, such as the relationship between *BRCA1* mutations and poly ADP-ribose polymerase (*PARP*)^1^, both of which are involved in DNA repair processes, or the recently identified interaction between *ARID1A* and *ARID1B*^30^. We therefore characterized MiSL predictions according to known cellular pathways (based on Kyoto Encyclopedia of Genes and Genomes^31^ or Gene Ontology^32^), to determine whether the MiSL candidates of a mutation shared pathways with the mutated gene. We use breast cancer as an illustrative example, where we found that the MiSL candidates of five recurrent mutations were enriched for genes in the same pathways as the respective mutations (Supplementary Fig. 3a). Interestingly, MiSL candidates of *BRCA1* mutation in breast cancer were significantly enriched for DNA repair genes, including *XRCC6* and *FANCC*, with *P* value=0.006 (hypergeometric test, Supplementary Fig. 3a), consistent with the clinically established SL interaction between inhibition of DNA repair and *BRCA1*mut^1^,^33^. We also found same pathway enrichment for other breast cancer mutations: ubiquitination genes for *MAP3K1*mut (*P* value=2.3 × 10^−6^), growth factor signalling (specifically neurotrophin, including AKT signalling) for *PIK3CA*mut (*P* value=0.0002), ‘response to endogeneous stimuli' for *GATA3*mut (*P* value=0.02), and adherens junction genes for *CDH1*mut (*P* value=0.02). Similarly, we observed same pathway enrichment for multiple mutations in nine other cancers (Supplementary Data 4). Furthermore, MiSL candidates were enriched for druggable pathways as per DGidb^34^ in multiple cancer types (Supplementary Data 5), suggesting that MiSL can identify mutation-specific druggable SL pathways in each cancer type.

We also noted that some MiSL candidates were shared by multiple mutations and multiple cancers, suggesting that certain genes are prone to synthetic lethality. We identified genes that were MiSL candidates for >5% of the MiSL-targetable mutations in each cancer type (cancer-specific recurrent MiSL candidates Supplementary Fig. 3b) and also identified genes categorized as MiSL candidates across cancer types (pan-cancer recurrent MiSL candidates). Out of the 7,257 genes predicted to be a MiSL candidate for at least one mutation in the 12 cancer types, 1,005 genes were candidates for ≥1 mutations in at least 6 different cancer types (Supplementary Data 6). We found pathways enriched among cancer-specific and pan-cancer recurrent MiSL candidates (Supplementary Data 6) including Krebs cycle, DNA repair and Wnt pathway, suggesting that targeting these pathways can provide new treatment options for many patient subgroups in different cancers.

### MiSL identifies a novel druggable target for IDH1 mutation

**Figure 3:**
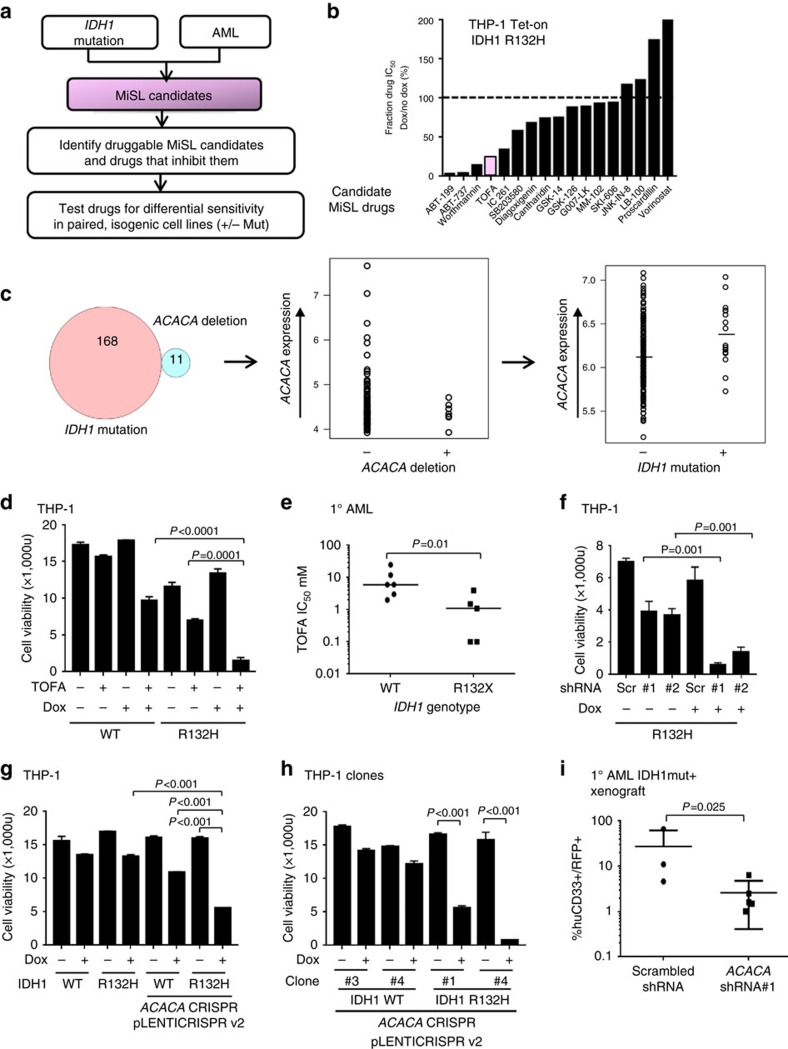
MiSL identifies a novel therapeutic target for the IDH1 mutation in acute myeloid leukaemia. (**a**) Schematic for testing druggable targets for IDH1 R132H mutation in AML. MiSL candidates were filtered for druggability using the DGIdb database. Seventeen compounds were obtained that were predicted to antagonize/inhibit the products of these genes, which were tested for synthetic lethality with IDH1 R132H. (**b**) Seventeen drugs were tested in the absence (−Dox) or presence (+Dox) of IDH1 R132H mutation and IC_50_s were calculated based on cell viability after 72 h. (**c**) MiSL found (i) mutual exclusion of *IDH1* mutation and *ACACA* gene deletion, (ii) *ACACA* deletion causes low expression (*P*=0.001) and (iii) expression of *ACACA* is higher in *IDH1*-mutated AML (*P*=0.008). (**d**) Viable cell growth (PrestoBlue fluorescence) of THP-1 cells at 10 days expressing IDH1 wild type (−Dox) or R132H mutant (+Dox) plated in 2 μM TOFA or DMSO in 0.5% serum, bars show s.d., *P* value=0.0001. (**e**) Purified primary *IDH1* mutant and *IDH1* wild-type AML blasts plated in increasing concentrations of TOFA. Sigmoidal dose–response IC_50_s were calculated and compared with Mann–Whitney *U*, *P* value=0.01. (**f**) Viable cell growth of THP-1 cells expressing IDH1 wild type (−Dox) or R132H mutant (+Dox) transduced with scrambled or ACACA shRNA lentivirus in 0.5% serum as in **d**. (**g**) Viable cell growth of THP-1-inducible IDH1 wild-type or R132H (+/−Dox) cells transduced with lentiviral pLENTICRISPR v2 targeting *ACACA* exon 4 versus non-*ACACA* targeted controls. (**h**) Growth of single clones from the same pLENTICRISPR v2 transduced THP-1 cells as in **g**. (**i**) Primary *IDH1* R132 mutant AML blasts were transduced with *ACACA* shRNA #1 or scrambled shRNA and transplanted into immunodeficient NSG mice. Bone marrow at 12 weeks was analysed for human CD45^+^CD33^+^ RFP^+^ cells. Scatter plot shows absolute and average percentage of hCD33^+^ RFP^+^ cells gated on human CD45^+^ engrafted cells (*n*=3, non-targeting versus *n*=5, shRNA#1, *P<0.05, Mann–Whitney). Panels **d** and **f**–**h** show representative of three independent experiments using four biological replicates; Student's *t*-test is used to calculate significance with *P* values as shown.

Next, we sought to use MiSL to discover a novel and druggable mutation-specific target. We focussed our efforts on the *IDH1* mutation in AML, as an example of a high-frequency mutation not represented in cell lines. We identified the druggable MiSL candidates and tested reagents that inhibited the genes for selective activity against *IDH1* mutant AML (Fig. 3a, Supplementary Fig. 4a). Seventeen out of the 89 MiSL candidates for *IDH1* mutation in AML were druggable using available reagents in the clinic or under development. Dose–response IC_50_ curves were generated in the presence (+dox) and absence (−dox) of IDH1-R132H in the inducible THP-1 cells^27^ for each drug (Fig. 3b). We noted a selective reduction in cell viability with the small molecule 5-(tetradecycloxy)-2-furoic acid (TOFA), a selective competitive inhibitor of acetyl-CoA carboxylase 1 (*ACACA*)^35^, which MiSL predicted to be a SL partner of the *IDH1* mutation in AML: there was a strong HI-LO Boolean implication between *IDH1* mutation and *ACACA* deletions in the pan-cancer TCGA data with zero overlap between the two events, *ACACA* deletions resulted in lowered expression of *ACACA*, and *ACACA* was differentially overexpressed in *IDH1*-mutant AML compared to *IDH1*-wild-type AML (Fig. 3c). Importantly, no difference in TOFA IC_50_ was observed in THP-1 cells transduced with tet-on IDH1 wild type tested against the same panel (Supplementary Fig. 4b). Pharmacological inhibition of ACACA with TOFA (Fig. 3d) caused a reduction in cell growth in the presence of IDH1 R132H (+dox), but not in its absence (−dox, *P*=0.0001, Student's *t*-test) or compared to IDH1 wild-type induction consistent with the drug screen (+dox, *P*<0.0001, Student's *t*-test). The growth defect of IDH1 R132H THP-1 cells was noted at only 2 μM TOFA, at the lower end of the reported IC_50_ range. Additionally, primary IDH1 R132-mutated purified AML blasts showed a 1.5–2.0-fold increase in *ACACA* gene expression compared to CD34^+^ hematopoietic stem and progenitor cells (Supplementary Fig. 4c,d) and were selectively sensitive to TOFA treatment compared to IDH1 wild-type AML blasts (IC_50_ 1.1 versus 6 μM, *P* value=0.01, Mann–Whitney) (Fig. 3e).

To specifically validate *ACACA* as a SL partner of *IDH1* mutation, we used two orthogonal gene-targeting methods: First, knockdown of *ACACA* with two independent shRNAs (Fig. 3f, Supplementary Fig. 4e) caused a defect in cell growth in the presence of IDH1 R132H (+dox) but not in its absence (−dox) or with scrambled shRNA (*P* value=0.001, Student's *t*-test, shRNA#1 IDH1 R132H +dox versus −dox; *P* value=0.001, Student's *t*-test, shRNA#2 IDH1 R132H +dox versus−dox). Notably, shRNA#3 which did not show on-target activity for ACACA (Supplementary Fig. 4e) also did not impair growth (Supplementary Fig. 4f). Dox treatment alone on parental THP-1 cells showed no effect on cell growth (Supplementary Fig. 4g). Second, THP-1 cells transduced with CRISPR/Cas9 targeting exon 4 of *ACACA* (Supplementary Fig. 4h–k) showed a growth defect in the presence of IDH1 R132H (*P* value<0.001, Student's *t*-test) but not IDH1 wild type both as cell pools (Fig. 3g) and for independent clones selected for the presence of indels (Fig. 3h, Supplementary Fig. 4l). Finally, primary IDH1-mutant AML cells transduced with shRNA#1 lentivirus targeting *ACACA* exhibited reduced engraftment of red fluorescent protein (RFP)-positive human CD45^+^CD33^+^ leukaemic cells compared to scrambled non-targeting shRNA (*P* value=0.025, Mann–Whitney) in immunodeficient NSG xenografts (Fig. 3i, Supplementary Fig. 4m).

Together, these data suggest that *ACACA* is a *bona fide* SL partner for the *IDH1* mutation in AML with direct clinical implications. Additionally, this validates MiSL's ability to identify new mutation-specific SL partners that are cancer specific and indicates that the methodology used here (Fig. 3a) can be used to identify novel pharmacological targets.

### MiSL identifies genetic biomarkers for targeted therapies

**Figure 4:**
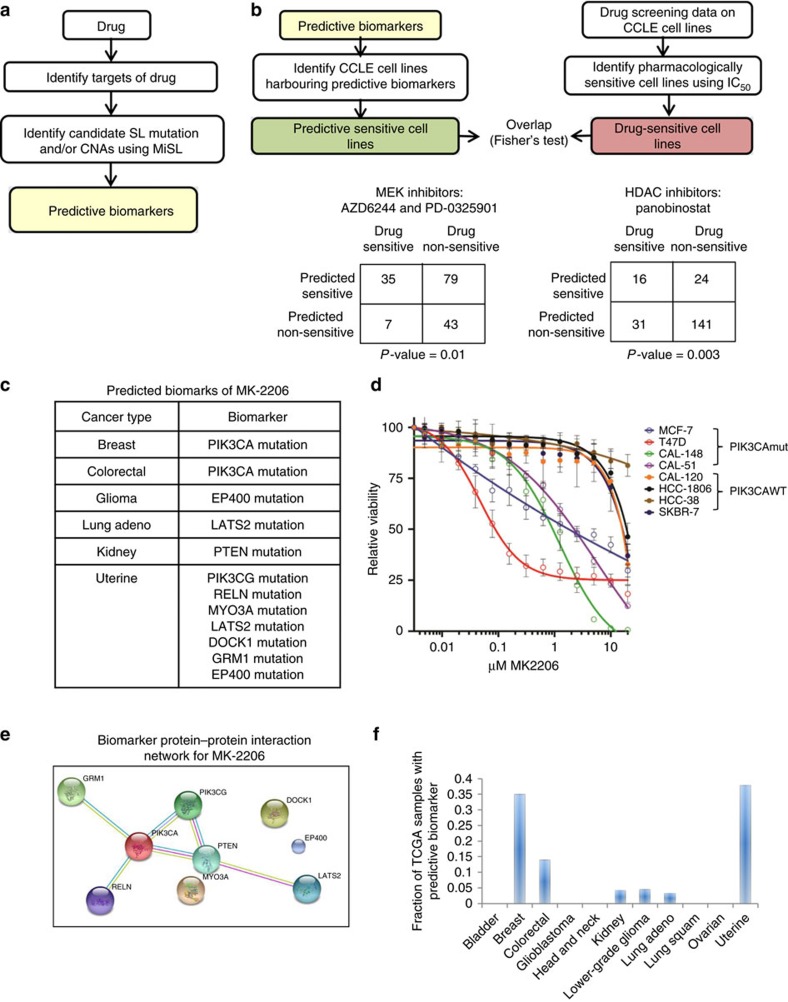
MiSL identifies predictive genetic biomarkers for existing targeted therapies. (**a**) Pipeline showing the use of MiSL to identify predictive biomarkers for a drug. The predictive biomarkers could be gene-specific mutations or CNAs in a particular cancer. (**b**) Validation of MiSL biomarker predictions using pharmacological data for CCLE cell lines. For a given target family (such as MEK or HDAC), cell lines from the CCLE that harbour the biomarkers identified by MiSL were predicted to be sensitive, and cell lines in the first quartile (based on IC_50_ values) were considered to be pharmacologically sensitive. Overlap analysis between the cell lines predicted to be sensitive by MiSL and the pharmacologically sensitive cell lines as per IC_50_ data shows a statistically significant overlap (MEK inhibition—*P* value=0.01; HDAC inhibition—*P* value=0.003, Fisher's exact test). (**c**) Predictive biomarkers as identified by MiSL are listed for the AKT1 inhibitor MK-2206. Besides *PIK3CA* mutation in breast cancer, several mutations in colorectal, lung adeno, kidney and uterine cancer were identified. (**d**) *PIK3CA* mutant (MCF-7, T47D, CAL-148, CAL-51) and *PIK3CA* wild-type (CAL-120, HCC-1806, HCC-38, SKBR-7) breast cancer cells were plated in the presence of increasing concentrations of Akt1 inhibitor MK-2206 and viability was measured at 72 h using CellTiter-Blue. (**e**) Several of the altered genes are related to *PIK3CA* as per STRING protein–protein interaction analysis. (**f**) Fraction of samples among the 12 TCGA cancers with MiSL-identified predictive biomarkers for the Akt1 inhibitor MK-2206.

Identification of predictive biomarkers for stratifying and assigning patients to targeted therapies is an area of active investigation in oncology^36^. Previous work has focussed on developing predictive models using drug-sensitivity data from cell lines^37^,^38^,^39^. MiSL provides an alternate synthetic lethality-based approach to identify predictive biomarkers for targeted therapies. Specifically, MiSL can identify mutations and/or CNAs in specific cancers that are SL partners of the gene products inhibited by a given drug (Fig. 4a), which then function as predictive genetic biomarkers for the drug. To test this, we compared MiSL-based predictions of sensitive cell lines with pharmacological data available for the CCLE^38^, which spans data for 24 compounds (targeted and cytotoxics) across 479 cell lines. To maximize the number of cell lines with pharmacological data, we grouped inhibitors of a target family, such as histone deacetylase (HDAC) inhibitors. Next, we used DGidb^34^ to identify genes whose products were inhibited by the drug(s). For example, for HDAC inhibitors, DGidb identified 14 genes (Supplementary Fig. 5a). MiSL then identified mutations and/or CNAs in each cancer type that were SL partners of these inhibited genes. Specifically, the genes inhibited by the drug were MiSL candidates of the identified mutations and/or CNAs. Cell lines that harboured these MiSL-identified biomarkers were predicted to be sensitive to the inhibitor.

To identify ‘true' sensitive cell lines, we used pharmacological data for the drug in question. A cell line was considered to be truly sensitive to a drug if it was in the first quartile of all tested cell lines based on IC_50_ values for the drug. Based on drugs with known target information as per DGidb and the number of ‘true' sensitive cell lines, there were five evaluable target families: mitogen-activated extracellular signal-regulated kinase (MEK), HDAC, RTK, RAF, and EGFR (Supplementary Fig. 5b). We found a statistically significant overlap between cell lines that were predicted to be sensitive based on the presence of MiSL-predicted biomarkers and those that were truly sensitive for multiple target families (MEK, HDAC) (Fig. 4b, Supplementary Data 7, Supplementary Fig. 5c), demonstrating potential applicability of MiSL to identify predictive genetic biomarkers. Notably, MiSL identified several genetic biomarkers not represented in existing cell lines. These included *LAMA3* mutations (lung squamous) and *NOS1* mutations (breast cancer), which were identified as MEK inhibitor-specific biomarkers, and *ST18* mutations (lung adenocarcinoma and ovarian cancer), which were identified as HDAC inhibitor-specific biomarkers.

Next, we applied MiSL to identify predictive biomarkers for an existing targeted drug that has clinical activity as monotherapy. We focussed on MK-2206, a selective inhibitor of *AKT1* in clinical trials in solid tumours^40^,^41^. MiSL identified several predictive biomarkers for MK-2206 (Fig. 4c), including *PIK3CA* mutation in breast cancer, which was identified because *PIK3CA* mutation and *AKT1* deletion were mutually exclusive in pan-cancer data, *AKT1* deletion resulted in lowered expression and *AKT1* was overexpressed in *PIK3CA*-mutant breast cancer (Supplementary Fig. 5d). To confirm MiSL's prediction, we treated a panel of *PIK3CA* mutant (MCF-7, T47D, CAL-148, CAL-51) and *PIK3CA* wild-type (CAL-120, HCC-1806, HCC-38, SKBR-7) breast cancer cell lines with MK-2206 in 5-day cell viability assays (Fig. 4d) and colony-formation assays (Supplementary Fig. 5e). Significantly, we found all four *PIK3CA* mutated breast cancer cell lines were sensitive to MK- 2206 (IC_50_<1 versus >15 μM, *P* value=0.003, Mann–Whitney) in both viability and colony assays (Fig. 4d, Supplementary Fig. 5e) with increased cleaved PARP and decreased phospho-PRAS40, S6K, 4EBP1 and BAD (Supplementary Fig. 5f). MiSL also identified predictive biomarkers for MK-2206 in other cancer types (Fig. 4c). Several predictions involved mutations in genes functionally associated with PIK3CA-AKT1 signalling (including *PTEN*mut in kidney cancer, *LATS2*mut in lung adenocarcinoma and *PIK3CG*mut in uterine cancer), suggesting that the method identifies biologically meaningful biomarkers (Fig. 4e). Further analysis indicated that 3–37% of TCGA samples in 5 different cancer types could be matched to MK-2206 based on MiSL-predicted biomarkers (Fig. 4f), demonstrating the potential of using MiSL to broaden the indications of existing anticancer therapies and identify patients who might benefit.

## Discussion

A crucial challenge in precision medicine is the identification of mutation-specific therapies for different cancers. We have developed MiSL, a simple and scalable Boolean implication-based computational method that analyses mutation, copy number and gene expression data of primary tumours to identify SL partners of specific mutations in specific tumour types. Extensive validation for multiple mutation and cancer combinations using both existing data and our own large-scale shRNA data confirmed that MiSL is an *in silico* screen that enriches for SL interactions (Fig. 2).

We envision MiSL as part of a larger process in which a tractable list of candidate targets (say, 20–200) is first identified computationally and then these targets are validated in depth. We tested this vision by setting out to find a novel SL partner of the *IDH1* mutation in AML and succeeded by discovering that *ACACA* is such a partner, validated *in vitro* and *in vivo* (Fig. 3d–i). *ACACA* is one of very few purported SL partners of recurrent tumour mutations to have been validated *in vivo*. This finding could be relevant to other *IDH1* mutant tumour types. Besides AML, *IDH1* mutation is present in 77% of lower-grade glioma and 7% of glioblastoma in the 12 cancers we analysed. MiSL also predicted *ACACA* to be a SL partner of the *IDH1* mutation in glioma: along with the mutual exclusion between *IDH1* mutation and *ACACA* deletions in pan-cancer data, *ACACA* was found to be overexpressed in glioma samples with the *IDH1* mutation as compared to *IDH1* wild-type samples (*P* value=0.008, Student's *t*-test). Given MiSL's prediction in AML and glioma and the positive validation of the *IDH1*mut-*ACACA* SL relationship in AML, it seems plausible that *IDH1*mut-*ACACA* SL relationship is valid in other *IDH1* mutant tumours. Selective inhibitors for acetyl CoA carboxylase are currently in development for the treatment of several metabolic diseases^42^. Our results suggest that they may have antiproliferative activity in *IDH1* mutant cancers, including AML, glioma, secondary glioblastoma and osteosarcoma. We have also shown that MiSL can be used successfully in reverse, which is to identify predictive biomarkers (mutations and/or CNAs) for existing targeted therapies in specific tumour types (Fig. 4b,c), and experimentally validated a MiSL-identified predictive biomarker, *PIK3CA* mutation in breast cancer, for an existing targeted therapy, AKT1-inhibitor MK-2206 (Fig. 4d).

Recently, a computational method termed DAISY^21^ has been described that can predict SL interactions using tumour genomic data and cell line shRNA data. MiSL has important differences with DAISY, even though both methods identify an initial set of candidates using tumour genomic data and apply subsequent filtering to minimize false positives, leading to DAISY failing to identify many of the SL interactions described here. The differences can be understood by asking why DAISY does not identify any of the SL interactions we have validated: *IDH1*mut-*BCL2/BCL2L2*, *VHL*mut-*GLS*, *IDH1*mut-*ACACA*, and *PIK3CA*mut-*AKT1*. DAISY's first inference strategy, ‘genomic survival of the fittest', which looks for mutual exclusion, considers a small number of inactivating mutations (nonsense and frame-shift mutations), while MiSL handles all types of mutations. Since *IDH1* mutations are mainly missense mutations, this step would fail to identify *IDH1*mut interactions. The second inference strategy uses cell line shRNA screens, which prevents DAISY from identifying SLs for recurrent mutations that are not well represented in available cell lines or for tumours for which very few cell lines have been isolated. This DAISY step would miss all predictions related to the *IDH1* mutations because *IDH1* mutation is rarely present as an endogenous mutation in cell lines. The third inference strategy requires SL pairs to have correlated expression (measured by Spearman correlation coefficient *ρ*≥0.5): this step would miss all the interactions we validated as all these pairs fail this condition (the Spearman correlation coefficient for each of these is <0.25). This demonstrates that DAISY removes many true SL interactions. We next sought to determine whether DAISY's predictions are enriched for mutation-specific SL partners for the *IDH1* mutation in AML. Towards that end, we reimplemented DAISY using the first and third inference strategies (we excluded the second inference strategy since it requires shRNA data not available for the *IDH1* mutation) and applied it to the *IDH1* mutation. We compared the SL partners found by DAISY with those identified in our shRNA screen for mutant *IDH1* in AML (Supplementary Fig. 6a) and found that there was no statistically significant association between the two lists (Supplementary Fig. 6b,c), indicating that DAISY's predictions are not enriched for *IDH1*mut SL partners in AML. In contrast, MiSL's predictions were highly enriched for *IDH1*mut SL partners in AML (Fig. 2f, Supplementary Fig. 2f), demonstrating MiSL's ability to identify candidate mutation-specific SL partners that are enriched for true positives in a cancer type.

MiSL has several important features that increase its applicability to precision medicine. First, MiSL is ‘mutation-centric' in conception such that its focus on identifying SL partners of recurrent somatic mutations lends itself directly to clinical application. Increasingly, sequencing and somatic mutation data for common mutations are available to the clinician for a given patient's tumour. A recent outcome analysis of 570 targeted agents found that personalized therapy using a known genomic biomarker had a higher response rate and prolonged overall survival compared with a protein biomarker^43^, justifying a ‘mutation-centric' approach. Second, MiSL does not require functional data from cell lines, which allowed it to identify SL partners for mutations, such as *IDH1*, that are not well represented in cell lines. Additionally, the use of primary tumour data allows MiSL to capture *in vivo* tumour evolutionary relationships that may not be present in cell line data. Our analysis demonstrates that mutual exclusion and subset relationships between somatic mutation and CNAs in human cancer could indeed arise due to synthetic lethality effects. By using Boolean implications, which represent stringent statistical mutual exclusion and subset relationships^24^, MiSL reduces the number of false positives and enriches for true SL partners (Fig. 2f, Supplementary Fig. 2f). MiSL also benefits from using pan-cancer data: when a gene is mutated in multiple tumour types, MiSL uses all available tumour types to infer SL partners of the gene. For example, even though AML copy number data was not included for our analysis (due to lack of availability), MiSL identified candidate SL partners for AML mutations because those mutations occurred in other tumour types. Finally, we note that the three experimentally validated examples presented here involve metabolic processes (*PIK3CA*mut-*AKT1*, *IDH1*mut-*ACACA*; *VHL*mut-*GLS*), particularly pathways that are known to be perturbed in cancer metabolism^44^. MiSL may be useful in finding mutation-specific metabolic dependencies, which might not be easily identifiable using cell line screens.

Several avenues of future investigation stem from this work. It would be important to test *ACACA* inhibitors in other *IDH1* mutant cancers and design combination therapies for *IDH1* mutant cancers using *ACACA* inhibitors and mutant-*IDH1* inhibitors (such as AG-120). Finally, it would be useful to extend MiSL to identify SL combinations (gene pairs or groups), whose combined knockdown would be deleterious with a particular mutation.

## Methods

### Data preparation

MiSL uses data from 12 different TCGA cancers (Supplementary Fig. 1a). For each of these cancers, we used the mutation, copy number and gene expression data. The only exception is AML, where many samples did not have copy number data. The starting point of our analysis is the level 3 data downloaded from the TCGA website.

The processing of the level 3 TCGA data is:

*Mutation*. The mutation data specify the mutated genes and the mutation type on a per sample basis. A Boolean variable is introduced for each mutated gene. Boolean variables are also introduced for each type of mutated gene (such as frame-shift deletions, missense mutations, nonsense mutations and splice site mutations). For each sample, the Boolean variable associated with a given mutation is set to high if the mutation associated with the variable is present and to low otherwise.

*Copy number alteration*. The data for CNAs contain the segmented copy number data for the tumour and normal samples. For both types of samples, segments, where the absolute magnitude of the segment mean was >0.3, were retained. Furthermore, only segments with ≥5 markers were retained to remove regions with low-confidence output. Next, for each tumour sample, the tumour-specific alterations were determined by removing segments that had >50% overlap with altered regions in the corresponding normal sample. The remaining segments were used to find, in each tumour sample, the genes affected by a CNA. The hg19 assembly was used to identify the genes. Next, two Boolean variables are introduced—one for gene amplification and another for gene deletion. For each sample, the Boolean variables for amplification or deletion are high if the gene was found to be amplified or deleted (absolute magnitude of segment >0.3).

*Gene expression*. For microarray data, the data was normalized using the standard Robust Multi-chip Analysis algorithm^45^. For RNAseq data, the reads per kilobase of exon per million reads mapped values were log transformed. RNAseq data was primarily used for our analysis except for cases where there was limited RNAseq data.

### MiSL algorithm

Given a mutation *X* and a cancer type of interest, the MiSL algorithm consists of the following steps: First, all the cancers in which the mutation is present in at least 2.5% of the samples were identified. Next, genes were identified that had more copies in the presence of a mutation as determined by using Boolean implications^23^,^24^. Boolean implications between pairs of variables were detected using a statistical test consisting of two parts: first, Fisher's exact test was used to test dependence, then sparseness of a specific quadrant was tested by using a maximum-likelihood estimate of the error rate for the points in the sparse quadrant. An implication was considered significant if the *P* value from the Fisher test was less than a cutoff threshold (always <0.05) and the error rate was <0.1. The cutoff was chosen to obtain an acceptable false discovery rate. In this work, the cutoff was set such that the false discovery rate <0.05 (as calculated by the procedure described in previous work^24^). The implication extraction procedure was augmented for genomic alterations by adding artificial normal samples for the HI-HI implication extraction. In both data sets, a few genomic alterations existed that were present in almost all tumour samples. In order to find HI-HI implications involving these alterations, artificial normal samples (which do not harbour the mutations or CNAs) were added when deriving the implications between genomic alterations. This was an acceptable procedure since, unlike DNA methylation or gene expression, the mutations and chromosomal alterations were very likely to be cancer specific because germline mutations and CNVs (copy number variations) have been removed by TCGA.

A critical filtering step during the extraction of Boolean implications was to exclude genes that are merely passengers in large chromosomal alterations. This was done by restricting the search for Boolean implication for a particular alteration to tumour types where the presence of the particular alteration resulted in concordant changes in gene expression. A deletion in gene *A* was considered to be a passenger in a tumour type if *A* was not differentially downregulated (as per *t*-test with fold difference >1.2, *P* value <0.05) in samples with deletions in *A* versus the remainder of the samples. Similarly, amplification in gene A was considered to be a passenger in a tumour type if *A* is not differentially upregulated in samples with amplification of *A*.

Finally, the resulting gene set was filtered to only include genes that are differentially overexpressed in the presence of the mutation versus the wild type in the cancer of interest (as per *t*-test, *P* value <0.05).

*Code availability*. The code and data used for MiSL are available at the Stanford Digital Repository https://purl.stanford.edu/ny450yx7231.

*Data availability*. The data sets analysed in the study are available at the Stanford Digital Repository (https://purl.stanford.edu/ny450yx7231). All data generated during this study are included in this published article (and its Supplementary Information files).

### Comparison with Achilles colorectal data

For this analysis, shRNA data from Project Achilles (downloaded from https://www.broadinstitute.org/achilles)^25^ was used. The project used a library of 54,020 shRNAs targeting 11,194 genes using individual shRNAs that were lentivirally delivered to the cells. The abundance of the shRNAs was measured after the cells were propagated for 16 population doublings or 40 days in culture, whichever came first, and compared to the initial DNA plasmid pool. Subsequently, the data were normalized along with some quality-control steps based on replicate reproducibility and a measure of the overall distribution of shRNA normalized and logged read counts. The final output was a shRNA summary score for each cell line for all the shRNA that passed the quality-control steps. The shRNA summary score was defined to be log2-normalized ratio of the raw read value for the shRNA divided by the total raw read value for the replicates. Thus a lower shRNA summary score in a cell line implies greater dependence on the gene in that cell line. The table of shRNA summary scores for the 216 cell lines was the starting point of our analysis.

The first step in our analysis was to identify the evaluable mutations. Evaluability of a mutation was assessed as follows: (i) there were >5 cell lines with the mutation in the cancer type of interest, and (ii) there were >25 MiSL candidates for the mutation in the cancer type of interest for which shRNA data were available in Project Achilles. The former condition ensured we had enough mutated samples in a cancer type and the latter condition was a necessary requirement for our downstream analysis using GSEA, which requires the gene sets to be >25.

For an evaluable mutation, the analysis was done as follows. The first step was to filter out genes that had data for less than three shRNAs per gene. There were 10,967 genes that remained after the filtering. The goal was to rank the genes in terms of essentiality in colorectal cancer cell lines with a specific mutation versus wild-type samples. First, for each shRNA, a differential analysis was done using a *t*-test. Subsequently, for each gene, the shRNA with the lowest *P* value is picked. The score of the gene is equal to log10 (*P* value) weighted by the difference in means between the two groups for that particular shRNA. Thus a gene that is more essential is given a stronger positive score. The genes were ranked using the score. GSEA was performed using GSEAPreranked from the GenePattern website^46^. For GSEAPreranked, the number of permutations was set to 2,000. The ranked list of genes was compared to the MiSL predictions for the same mutation in colorectal cancer to perform enrichment analysis.

### SL IDH1 shRNA screen for MiSL validation

The IDH1 WT and R132H-inducible THP-1 cell lines were transduced with the DECIPHER 27K Pooled shRNA lentivirus libraries Human Module 1 and 3 (Cellecta), as these modules overlapped the most number of MiSL candidates. Each cell line used in the study was obtained from ATCC or DSMZ and identity was confirmed using short tandem repeat analysis (Bio-synthesis, Louisville, TX). Periodically cells were tested for mycoplasma contamination using enzyme-linked immunosorbent assay-based method (Roche Life Science, Indianapolis, IN). Each library contains 275,000 unique shRNA constructs targeting 5,043 human genes (approximately 5 or 6 redundant shRNAs per gene) in the pRSI9 shRNA expression vector. The vector contains the following elements: (i) U6 RNA polymerase III promoter driving shRNA expression, (ii) 18-nucleotide DNA barcode sequence and (iii) UbiC promoter driving RFP expression to mark transduced cells. For each inducible cell line, 12 million cells were transduced at an efficiency of 30–40% to ensure that ∼90% of the transduced cells were single integrants according to the Poisson distribution. The number of transduced cells was approximately 100-fold the complexity of the library. Three days after transduction, each cell population was divided into two flasks. Dox was added to one of the flasks at a concentration of 1 μg ml^−1^ to induce expression of either wild-type or mutant IDH1^R132H^. The cells were expanded and selected in culture for 12 additional days. During this period, the number of transduced cells in each flask was maintained at >1,000-fold the complexity of the library. After the selection period, the cells were centrifuged, and genomic DNA was extracted using a QIAamp Blood DNA Maxi Kit (Qiagen, Valencia, CA) and submitted to Cellecta, Inc. for bar code amplification, high-throughput sequencing and deconvolution. Twenty million barcode reads were performed for each sample.

The following method was used to identify SL hits from shRNA data. shRNA constructs with <100 barcode reads in the THP-1 R132H no-Dox sample were excluded for further analysis to minimize noise associated with inadequate baseline representation. Genes with less than three redundant shRNA were excluded. The remaining constructs that (i) had a >20% reduction in the number of reads in the presence of Dox compared to the number of reads in the absence of Dox in THP-1 R132H cells and (ii) had a mean drop-out ratio of <0.6 in the presence of Dox versus the absence of Dox were considered shRNA hits. Of the 8,189 genes in the libraries, 776 were considered SL hits using this method.

### Inhibition of GLS in VHL mutant cell lines

Paired RCC4 and RCC4+VHL cells (2,000 cells per well) were plated in DMEM containing 1 mM of glutamine in a 96-well plate. After 24 h, cells were treated with either 25 nM siGLS SMARTpool or siControl SMARTpool (Dharmacon). Media was changed 24 h after siRNA treatment; 48 h after changing the media, viability was determined using the CellTiter-Glo Assay (Promega). Percentage of viable cells was determined by normalizing the treatment to the different controls.

### Pathway enrichment

For pathway analysis, Kyoto Encyclopedia of Genes and Genomes and Gene Ontology BP gene sets from the MSigDB website were used. The MiSL candidates of a mutation *X* were said to be enriched for the same pathway if the following criterion were satisfied: (1) the mutated gene belongs to a pathway *P*, and (2) the MiSL candidates of *X* have a statistically significant overlap (*P*<0.05) with the genes in *P*. To get as specific pathways as possible, all pathways that had >500 genes were removed. Furthermore, to remove redundant results, certain pathways were filtered out according to the following criterion. If two pathways *P1* and *P2* found the same overlapping set, the pathway with the larger (worse) *P* value was removed. Similarly, if the overlap set for a pathway was completely contained in the overlap set of another pathway, the first pathway was removed.

### Breast cancer cell lines

Breast cancer CAL-120 and CAL-51 cell lines were cultured in DMEM; T47D, MCF-7, HCC-1806 and HCC-38 cells in Roswell Park Memorial Institute (RPMI) 1640; CAL-148 were cultured in Minimum Essential Media (MEM) supplemented with 1 μg per 100 ml EGF; and SKBR-7 cells were cultured in DMEM/F12 medium. Medium was supplemented with 1% glutamine, 1% penicillin/streptomycin and 8% FCS or 16% FCS (CAL-148 and CAL-51 cells).

### CellTiter-Blue viability assay

Breast cancer cells were seeded in a 384-well plate. After 24 h, inhibitor was added to the medium in twofold serial dilutions using a HP Direct Digital Dispenser. After 72 h of culture, CellTiter-Blue (Promega) was added. The conversion of resazurin into resofurin was measured by using an EnVision Multilabel Reader. Treatment with 10 μM phenyl arsenic oxide was used as a baseline for viability.

### Colony-formation assays

Breast cancer cells were seeded in six-well plates and cultured both in the absence and presence of MK-2206 as indicated. All cells were fixed with 4% formaldehyde and stained with 0.1% crystal violet when wells containing untreated cells became confluent.

### Protein lysate preparation and western blot analysis

Cells were lysed in RIPA buffer containing 150 mM NaCl, 50 mM Tris pH 8.0, 1% NP-40, 0.5% sodium deoxycholate and 0.1% SDS supplemented with protease inhibitors (Complete, Roche) and phosphatase inhibitor cocktails II and III (Sigma) and boiled for 10 min after addition of sample buffer (60 mM Tris pH 6.8, 5% glycerol, 1% SDS, 2% β-mercaptoethanol, 0.02% bromophenol blue) before SDS gel electrophoresis followed by western blotting. Primary antibody against HSP90 (SC-7947) was purchased from Santa Cruz. Antibodies against acetyl CoA carboxylase 1 (#4190), beta-actin (#4967), cl-PARP (#9542), p-AKT (#4060), p-PRAS40 (#2997), p-S6 (#2211), p-4EBP1 (#9456) and p-BAD (#5284) were from Cell Signaling. Secondary antibodies were obtained from Bio-Rad Laboratories and Thermo Scientific. Uncropped blots are shown in Supplementary Fig. 7.

### Predicting genetic biomarkers with MiSL

For a given drug, the set of genes that are inhibited by the drug using DGidb^38^ were identified. Assume that drug *D* inhibits a set of genes *S*. Subsequently, MiSL was used to determine which genomic alterations in a given cancer would be SL with the inhibition of each gene in S, say gene *Y*. This involved looking for HI-LO implications with deletion of *Y* and HI-HI implications with amplification in *Y*. Any alteration that was located in the same chromosome as *Y* was removed from consideration to minimize false positives. The search for implications was done in a pan-cancer manner in cancer types where alteration in *Y* is not a passenger (as determined by gene expression analysis described earlier). Subsequently, for each identified genomic alteration that had a Boolean implication in the previous step, filtering was done to remove alterations if gene *Y* was not overexpressed in the samples with the genomic alteration versus the wild-type samples in each cancer of interest.

### Comparing MiSL predictions with pharmacological data

The comparison pipeline was as follows: Given a target for inhibition (such as HDAC), we determined the drug (Panobinostat) that was used to inhibit it. Next, the genes that were inhibited by the drug were identified using DGidb^34^. Subsequently, MiSL was used to identify predictive biomarkers for the drug as described in the previous section. Next, cell lines that had the predicted biomarkers (either mutations and/or CNAs) were identified. This gave us the list of MiSL-identified sensitive cell lines. The pharmacological data was used to identify ‘true' sensitive lines. Cell lines of the following tissue types—central nervous system, kidney, intestine, lung, ovary, breast—were used, as the tissue types were common to both the TGCA cancers and had pharmacological data. All cell lines that were in the first quartile based on IC_50_ values of all tested cell lines for the drug were deemed to be ‘truly' sensitive. A similar analysis was done when there were multiple drugs for a given target (MEK and RAF).

### AML druggability screen

THP-1 cell lines were cultured in RPMI supplemented with 10% FCS, 100 μg ml^−1^ of penicillin and 100 μg ml^−1^ of streptomycin and 1 μg ml^−1^ puromycin. ABT-199 was purchased from ChemieTek (Indianopolis, IN). Cantharidin, digoxigenin, proscardillin, wortmannin, SB203580, TOFA, IC 261 and vorinostat were all purchased from Sigma. ABT-737, GSK-J4, GSK-126, G007-LK, MM-102, SKI-606 and JNK-IN-8 were purchased from Sellekchem (Houston, TX). LB100 (HY-18597) was obtained from MedChemExpress (MonMouth Junction, NJ). After 4 days of Dox induction (or control without Dox), cells were plated in RPMI 20% foetal bovine serum at 2 × 10^5^ ml^−1^ in 96-well plate with twofold dilutions of each drug performed in duplicate. At 72 h, cell viability was measured using a plate-reader after addition of 10% Presto Blue Cell Viability Reagent (ThermoFisher Scientific) at emission fluorescence 590 nm. IC_50_ curves were calculated for each drug in the presence and absence of Dox using GraphPad Prism 6.0 (dose response inhibition) and the difference in IC_50_ was plotted as a percentage of control (no dox). For *ACACA* validation, transduced THP-1 cells were induced with Dox for 3 days, then washed into low serum RPMI (0.5%) and cultured at low cell density for 7 days +/−TOFA before cell growth was measured with Presto Blue on a plate reader.

### Lentiviral expression vectors

Lentivirally transduced pools of cells were selected in 1 μg ml^−1^ puromycin. IDH1 wild type and R132H mutation were expressed in the pTRIPZ (Open Biosystems) tet-inducible lentiviral vector with green fluorescent protein encoded in the same open reading frame by T2A peptide.

### CRISPR short guide RNA design and cloning

A total of 20 bp oligonucleotide sequences targeting exon 4 of the *ACACA* locus were designed using the Desktop Genetics platform (Deskgen.com) and four target sequences were chosen based on Rule Set Number 2. Annealed synthetic DNA oligos for each target sequence were phosphorylated and cloned into pX330-U6-Chimeric_BB-CBh-hSpCas9 (gift from Feng Zhang, Addgene plasmid #42230). In-del mutation frequency was measured using TIDE (Tracking of Indels by Decomposition: https://tide.nki.nl) after nucleofection of 10^6^ K562 cells with 2 μg of plasmid DNA using the Amaxa Nucleofector II, program T-016. Genomic DNA was isolated after 3 days in culture and exon 4 of ACACA flanking the cut site was amplified using forward (TAGGATGCTAGGGAGGCAGA) and reverse (TGATGGCATCTGCTGGTAAA) primers with annealing temperature of 61 °C. The sgRNA sequence with the highest cutting efficiency (65%) (GGCTTGCACCTAGTAAAGCA) was cloned into lentiviral LentiCRISPRv2_tagRFP vector (gift from Feng Zhang, Addgene plasmid #52961).


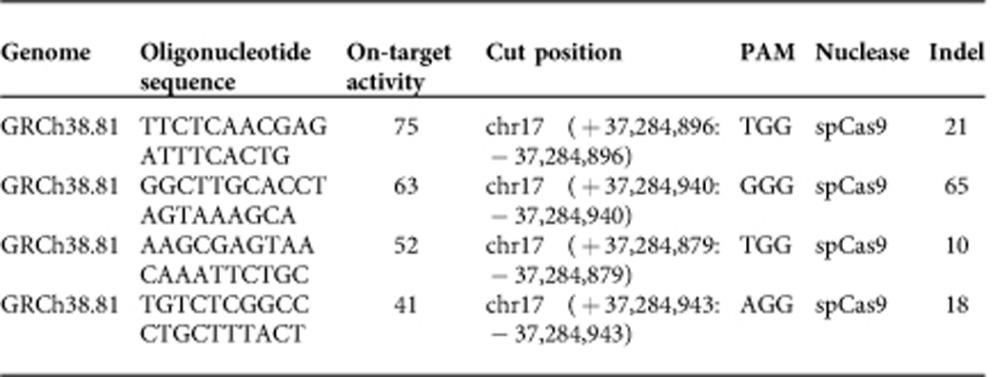


### Patient sample data

Primary bone marrow and peripheral blood AML samples were obtained with informed consent according to institutional guidelines (Stanford University Institutional Review Board No. 6,453 and No. 18,329). Mononuclear cells from each sample were isolated by Ficoll separation and cryopreserved in liquid nitrogen. All of the primary samples were tested for mutations in *FLT3*, *NPM1*, *IDH1* and *IDH2* by the Stanford Anatomic Pathology and Clinical Laboratories. *IDH*1/2 mutations were detected using SNaPshot methodology (Life Technologies). *ACACA* gene expression was determined using Taqman (Cat. #4,331,182, ThermoFisher) with *GAPDH* as a housekeeping control. AML blasts were cultured in OPTIMEM with 10^−6^ M hydrocortisone and 20 ng ml^−1^ each of interleukin-3, granulocyte macrophages colony-stimulating factor, granulocyte colony-stimulating factor, interleukin-6, stem cell factor, FLT3L and thrombopoietin (Peprotech).

### Animal care

All mouse experiments were conducted according to an Institutional Animal Care and Use Committee-approved protocol (Stanford Administrative Panel on Laboratory Animal Care no. 22,264) and in adherence to the US National Institutes of Health's Guide for the Care and Use of Laboratory Animals.

### Xenograft of Primary AML

Freshly thawed primary AML samples were transduced with lentivirus from pRSI9 DECIPHER shRNA expression vector (Cellecta, Mountain View, CA) on retronectin for 16 h and then one million cells were transplanted intravenously into 6-to-12-week-old NSG mice conditioned with 200 rad of irradiation. Both female and male mice were used. All mouse experiments were conducted according to an Institutional Animal Care and Use Committee-approved protocol (Stanford Administrative Panel on Laboratory Animal Care no. 22,264) and in adherence to the US National Institutes of Health's Guide for the Care and Use of Laboratory Animals. Up to five mice were transplanted for each treatment group if a sufficient amount of primary patient material was available, giving enough power to see a statistical difference of >30% Mann–Whitney *U*. A fewer number of mice were used if the sample was limiting. Mice were randomized per block prior to engraftment with either scrambled or ACACA shRNA. Investigators were not blinded to shRNAs transduced into AML after engraftment. Only IDH1mut AML samples were used in the study.

### Bone marrow engraftment analysis

Bone marrow cells were collected by aspiration of the femur using a 27-gauge needle and stained for 30 min at 4 °C with the following fluorophore-conjugated monoloncal antibodies: mTER199-PE-Cy5 (dilution 1:100; clone TER-199, eBioscience), mCD45-PE-Cy7 (dilution 1:50; clone A20, eBioscience), hCD45-V450 (dilution 1:25; clone HI30, BD), and hCD33-APC (dilution 1:25, clone WM53, BD). Viable cells were identified by propidium iodide exclusion. The human leukaemic population was identified as mTER199^−^, mCD45^−^, hCD45^+^ and hCD33^+^. Cells stably transduced with shRNA were identified as RFP^+^.

### Construction of ACACA shRNA expression lentiviral vectors

The human ACACA (GenBank accession code: NM_198834.2) shRNA target sequences were selected using the BLOCK-iT RNAi Designer tool (Life Technologies). Knockdown efficiency of ACACA shRNA constructs was determined by quantitative real-time PCR and western blotting. A pair of DNA oligonucleotides containing the sense target sequence followed by a loop sequence (5′-TCAAGAG-3′) and the reverse complement of the sense sequence were synthesized and annealed at 50 μM in annealing bugger (10 mM Tris-HCl pH 8.0, 50 mM NaCl, 1 mM EDTA) at 95 °C for 10 min, followed by a slow cooling over 1 h to room temperature. The double-stranded DNA template was then cloned into the pRSI9 DECIPHER shRNA expression vector (Cellecta, Mountain View, CA) digested with BsbI. The ACACA sequences targeted by shRNA vectors used in this study were 5′-UGGCAUUGCAGCAGUGAAA-3′(shRNA 1); and 5′-UGGAAUGAUUGCUGGAGAA-3′ (shRNA 2). shRNA 3 5′-GUGCUGGGACUGUGGAAUA-3′ was not found to be on-target.

### Statistical data analysis

Unless otherwise stated, *P* values comparing two means were calculated using the two-tailed unpaired Student's *t*-test in Prism version 6 (GraphPad Software, Inc. La Jolla, CA). For *in vivo* engraftment data, non-parametric Mann–Whitney *U*-test was used. A *P* value <0.05 was considered statistically significant. IC_50_ values were determined using the dose response (inhibition) function in Prism version 6.0. The data were normalized and fitted using a variable Hill Slope model.

### Comparison with DAISY

Since the DAISY paper^21^ does not include any predictions for missense mutations, we reimplemented DAISY as described in the paper and applied it to the *IDH1* mutation. As described in the paper, DAISY has three inference strategies: (1) Genomic survival of the fittest, (2) shRNA-based functional examination, and (3) pairwise gene co-expression. DAISY intersects the predictions from these three inference strategies to determine its list of candidates for a specific alteration. For steps 1 and 3, we used the same thresholds as outlined in the Methods section in the paper. Since the *IDH1* mutation is present in very few cell lines (<2%), the shRNA-based functional examination cannot be done for this particular mutation. Hence, we excluded step 2 from the analysis. Thus the list of DAISY candidates for the *IDH1* mutation was obtained by intersecting the results from steps 1 and 3.

### Data availability

All relevant data are available from the authors.

## Additional information

**How to cite this article:** Sinha, S. *et al*. Systematic discovery of mutation-specific synthetic lethals by mining pan-cancer human primary tumour data. *Nat. Commun.* **8**, 15580 doi: 10.1038/ncomms15580 (2017).

**Publisher's note:** Springer Nature remains neutral with regard to jurisdictional claims in published maps and institutional affiliations.

## Supplementary Material

Supplementary Information Supplementary Figures

Supplementary Data 1 Catalogue of MiSL-targetable Mutations and candidate MiSL candidates.

Supplementary Data 2 Number of MiSL candidates that are shared across different numbers of

Supplementary Data 3 Summary of MiSL pipeline parameters for all MiSL relationships.

Supplementary Data 4 Same pathway analysis for mutation-specific MiSL candidates in each cancer type.

Supplementary Data 5 Comprehensive pathway analysis for mutation-specific MiSL candidates in each cancer type.

Supplementary Data 6 Summary of Recurrent MiSL candidates (cancer-specific and pan- cancer) and pathway analysis.

Supplementary Data 7 Predictive Biomarker Analysis using MiSL.
