# Supplementary material for: Systematic discovery of mutation-specific synthetic lethals by mining pan-cancer human primary tumor data
Source: Nat Commun. 2017 May 31;8:15580. doi: 10.1038/ncomms15580 (PMC5460027; doi:10.1038/ncomms15580)
Supplement: Supplementary Information — Supplementary Figures [file ncomms15580-s1.pdf]

a

Cancer Type	# Samples with Mutation & Copy Number	# Samples with Mutation & Expression
Acute Myeloid Leukemia	-	181
Bladder	99	38
Breast	746	698
Colorectal	144	137
Glioblastoma	122	109
Head & Neck	291	248
Lower Grade Glioma	208	205
Lung Adeno	450	121
Lung Squam	157	157
Renal	388	354
Ovarian	308	261
Uterine	157	220

b

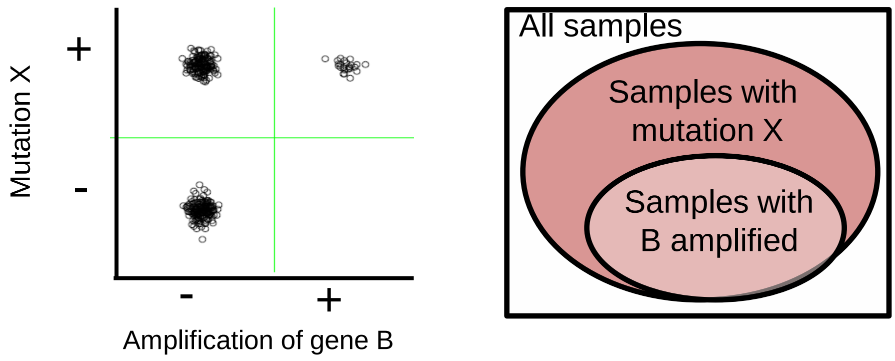

c

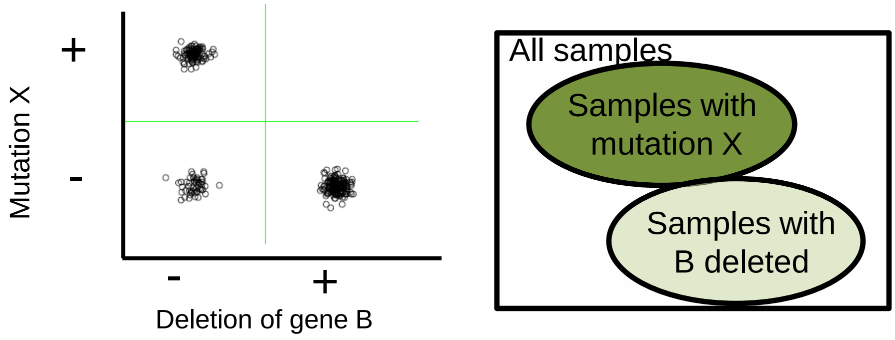

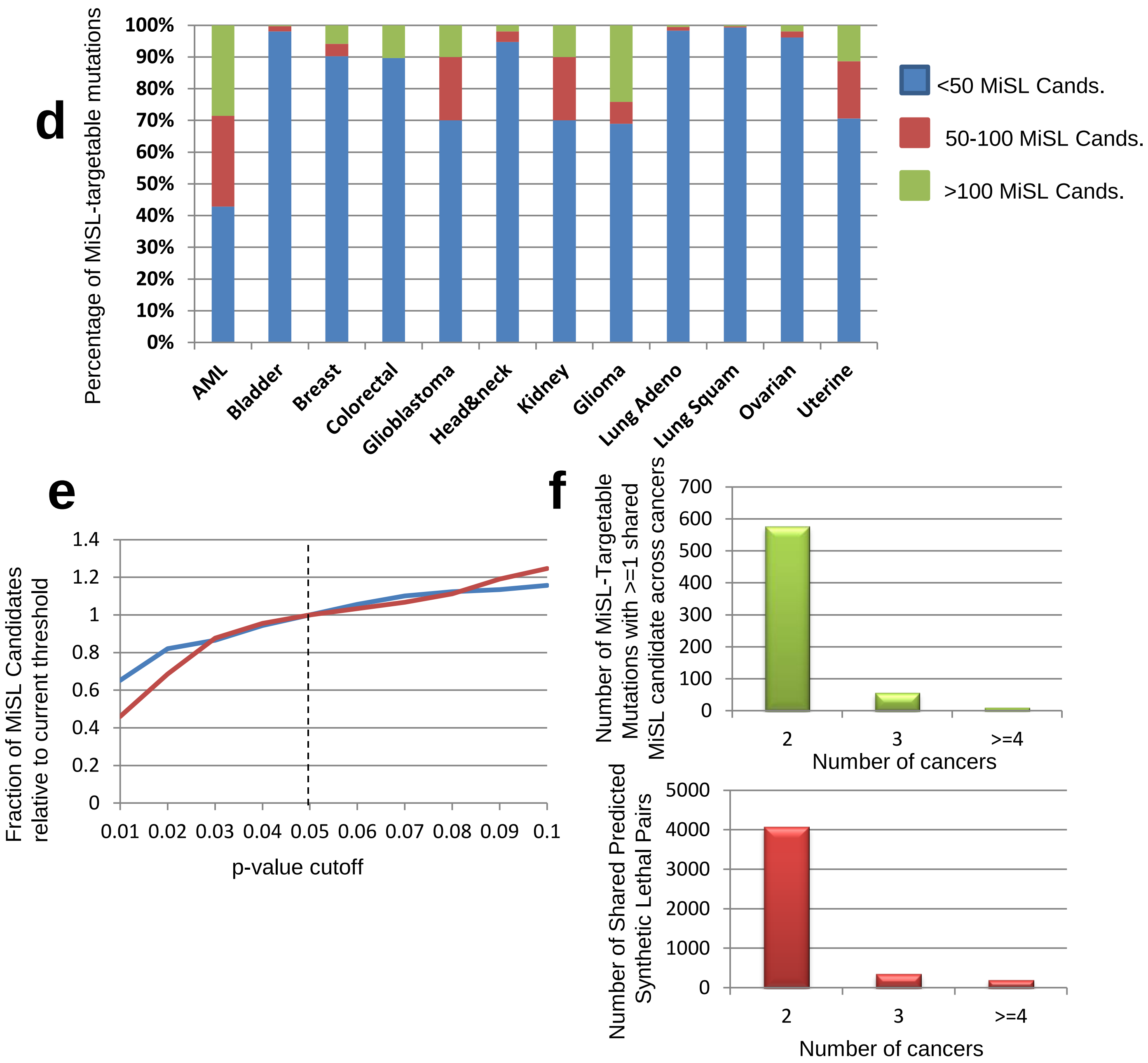

Supplementary Figure 1. Primary Tumor Samples and Cancer Types Used in MiSL. (a) Summary of Samples in 12 TCGA Cancers Analyzed. (b) HI-HI Boolean implication (if amplification B is present, then mutation X is present) represents a subset relationship between the two attributes. (c) HI-LO Boolean implication (if deletion B is present, then mutation X is NOT present) represents a mutual exclusion relationship between the two attributes. (d) Distribution of number of MiSL candidates for each MiSL-targetable mutation in each cancer type. (e) Sensitivity analysis by varying the p-value threshold for differential expression for copy number filter (blue) and expression filter (red) in MiSL pipeline (dashed line shows current threshold). (f) Distribution of shared synthetic lethal predictions across different cancers. The green barplot shown the number of MiSL-targetable mutations that have at least one common MiSL candidate in different number of cancers. The red barplot shows the number of synthetic lethal interactions (i.e., a {mutation, MiSL candidate} pair) that are common across different numbers of cancers.

a

Cancer	# of Evaluable Mutations	Evaluable Mutation
Breast	2	MAP3K1, PIK3CA
Colorectal	5	APC, CSMD3, KRAS, PIK3CA, TP53
Glioblastoma	1	PTEN
Kidney	0	-
Leukemia	1	TP53
Lung Squam	0	-
Ovarian	0	-

b

Mutation	p-value	NES
APC	0.003	1.55
CSMD3	> 0.1	-
KRAS	0.04	1.48
PIK3CA	0.03	1.35
TP53	>0.1	-
APC&KRAS	0.07	1.35

c

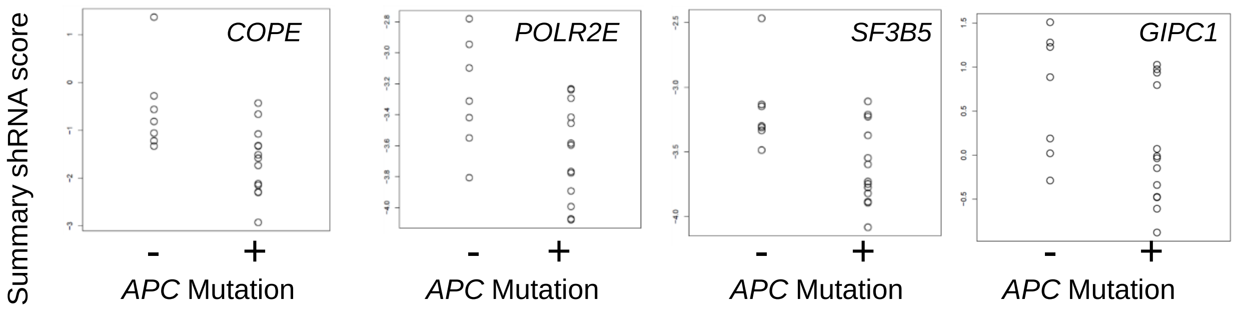

d

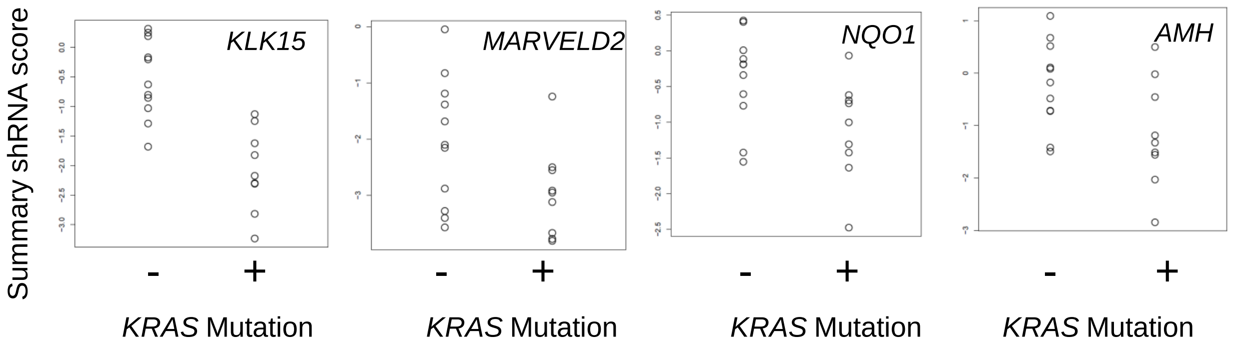

e

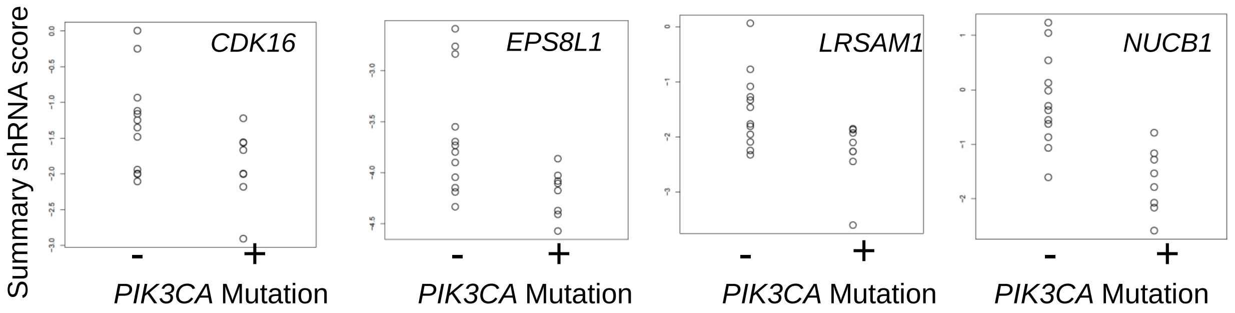

f

	shRNA Hits	shRNA Non-SL
MiSL Candidates	9	52
Not MiSL Candidates	430	7688

p-value = 0.004

g

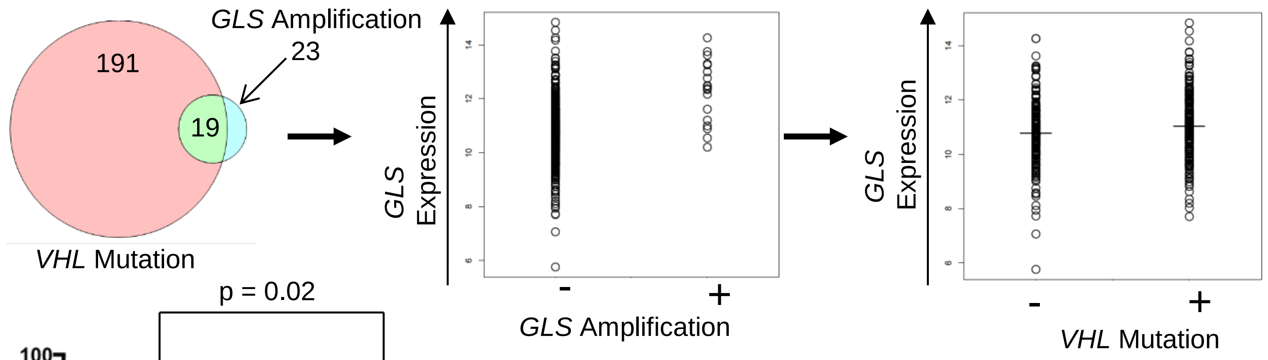

h

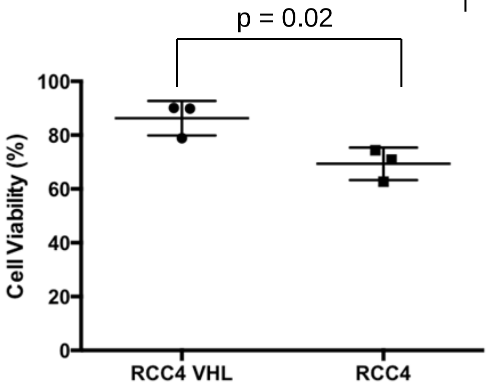

Supplementary Figure 2. Validation of MiSL. (a) Summary of Evaluable Mutations in Project Achilles for different cancers. (b) Summary of gene set enrichment analyses (normalized enrichment score and p-value) comparing enrichment of MiSL candidates in Achilles data for mutations in colorectal cancer. (c) Achilles shRNA summary scores for representative shRNAs in colorectal cell lines with *APC* mutation versus wild-type cell lines for 4 different MiSL candidates for *APC* mutation in colorectal cancer. (d) Achilles shRNA summary scores for representative shRNAs in colorectal cell lines with *KRAS* mutation versus wild-type cell lines for 4 different MiSL candidates for *KRAS* mutation in colorectal cancer. (e) Achilles shRNA summary scores for representative shRNAs in colorectal cell lines with *PIK3CA* mutation versus wild-type cell lines for 4 different MiSL candidates for *PIK3CA* mutation in colorectal cancer . (f) Contingency table showing overlap between MiSL candidates for *IDH1* mutation in acute myeloid leukemia and shRNA synthetic lethals as per DECIPHER screen with a more stringent criterion (drop-out ratio of 0.6 instead of 0.8 for 2 or more shRNAs) for calling synthetic lethals in shRNA data. The overlap is statistically significant with the more stringent criterion for calling synthetic lethals. (g) MiSL analysis steps illustrated for *VHL* Mutation and *GLS* – (i) subset relationship between *GLS* gene amplification and *VHL* mutation (HI-HI Boolean implication), (ii) amplification of *GLS* is concordant with higher expression of the gene ($p < 0.05$), (iii) expression of *GLS* is higher in *VHL*-mutated kidney cancer ($p < 0.05$). (h) Cell viability of RCC4 and RCC4/VHL after siRNA treatment to GLS. All error bars represent the SEM ($n = 3$) ($p = 0.024$).

a

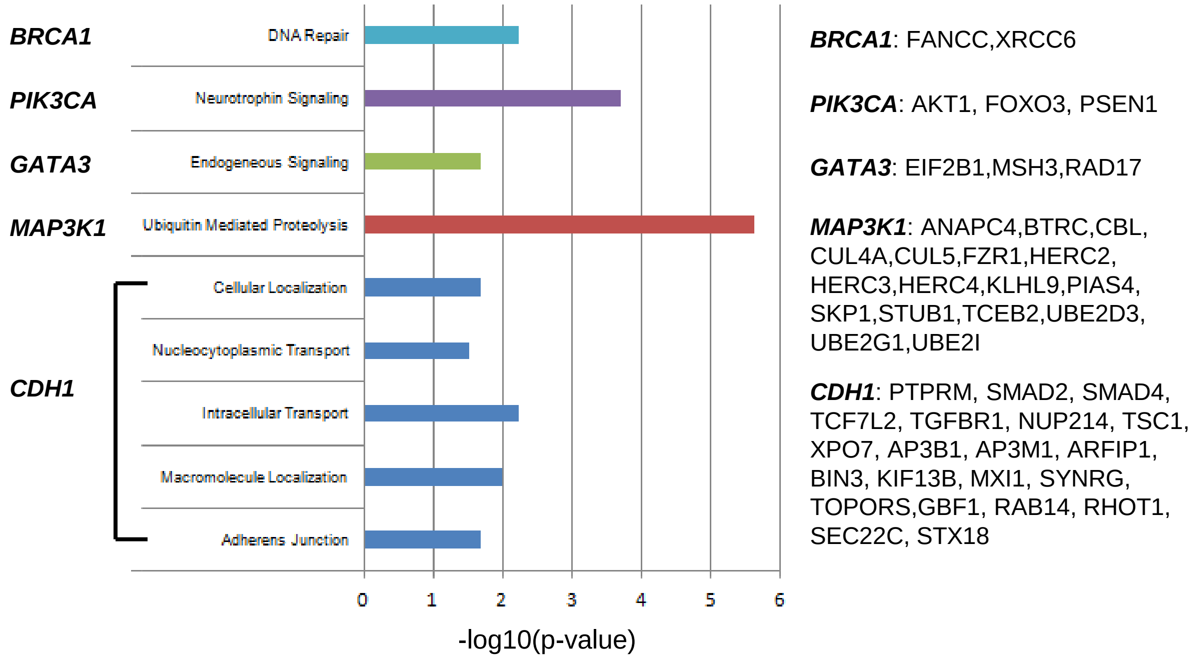

b

Cancer Type	# of MiSL candidates	# of Recurrent MiSL Candidates
AML	601	21
Bladder	1842	0
Breast	1758	56
Colorectal	1321	33
Glioblastoma	335	10
Head & Neck	1924	4
Kidney	299	16
Lower grade glioma	1794	420
Lung adeno	2772	0
Lung squam	2425	0
Ovarian	534	20
Uterine	4481	225

Supplementary Figure 3. Pathway Analysis of MiSL Candidates for Recurrent Mutations and Recurrent MiSL Candidates. (a) Same pathway enrichment analyses using GO and KEGG gene sets for MiSL-targetable mutations in breast cancer. The x-axis shows the negative log of p-value of Fisher's exact test. MiSL candidates are said to be enriched in the same pathway as the mutated gene if (i) the MiSL candidates of the mutation are enriched for a pathway where overlap is measured using Fisher's exact test (ii) the mutation belongs to the same pathway. Only statistically significant results ($p < 0.05$) are reported. (b) Recurrent MiSL Candidates in the Different TCGA Cancers. A MiSL candidate is called recurrent in a cancer if it appears as a MiSL candidate for more than 5% of the recurrent mutations in the cancer.

a

BCL2L2: ABT199,ABT737
PI4KA: Wortmannin
ACACA: TOFA
CSNK1E: IC261
NLK: SB203580
ZNF395: Digoxigenin
PPP2R2A, PPP2R2D: Canthardin
KDM6B: GSK-J4
SUZ12/SUZ12P- GSK-126
TNKS: G007-LK
MLL: MM-102
THRA: SKI-606
MAPK8: JNK-IN-8
PPP2R2A, PPP2R2D : LB100
ZNF248: Proscardillin
CLPX: Vorinostat

b

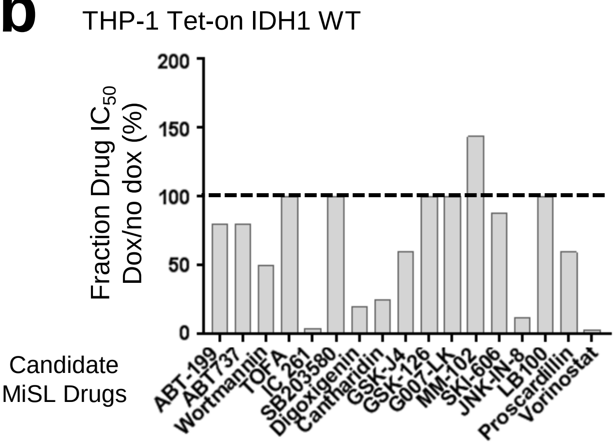

Supplementary Figure 4. Drug Screen for MiSL candidates of IDH1 mutation in AML. (a) List of druggable MiSL candidates for the IDH1 mutation in AML. (b) Fraction IC_{50} for each drug tested in inducible IDH1 wildtype cells comparing (+ dox) to (- dox) as a control. (c) Table showing clinical characteristics of *IDH1* mutant AML patient samples. (d) *ACACA* expression in sorted *IDH1* mutant blasts (n=8) and IDH1 wildtype blasts (n=5) from AML patients compared to normal CD34+ cord blood determined by Taqman expression assay. Bars=SD of 4 replicates. (e) *ACACA* mRNA expression for 3 different shRNAs designed to target *ACACA* determined by real-time quantitative PCR in triplicate after transduction of THP-1 cells with shRNA lentivirus expressed in pRSI9 virus. shRNA #3 showed minimal knockdown. Error bars represent standard deviations, * $p < 0.05$, unpaired t-test. ns = non-significant. (f) Viable cell growth of THP-1 pTRIPZ IDH1 R132H-T2A-GFP in 0.5% serum transduced with shRNA #3 showing minimal defect in presence of IDH1 mutation (+dox). (g) Cell growth of parental THP-1 cells treated with 1 μg/ml doxycycline in 0.5% and 10% serum. (h) In-del mutation frequency reported by TIDE from genomic DNA extracted from IDH1 wildtype and R132H inducible THP-1 cells transduced with pLENTICRISPR v2 encoding guide RNA targeting exon 4 of *ACACA*. This guide was selected out of four gRNA sequences that were designed and tested in K562 cells. (i) Sequencing of *ACACA* exon 4 showing disruption of *ACACA* gene in both IDH1 wildtype and R132H THP-1 cell pools. (j) Cell sorting strategy after lentiviral CRISPR transduction showing RFP+ THP-1 cells marked lentiCRISPR integration and doxyxcyline-inducible GFP-T2A-IDH1 wildtype and R132H. (k) Western blot showing reduced ACACA protein expression after CRISPR cutting in pooled cells compared to untransduced IDH1 WT or R132H inducible THP-1 cells respectively. (l) Sequencing of Exon 4 of *ACACA* from the single cell-derived THP-1 clones showing in/dels. (m) Flow cytometry showing engraftment of RFP+, CD33+ AML blasts in NSG mice, gated on engrafted CD45+ cells non-targeting shRNA vs *ACACA* shRNA #1.

c

AML No.	Age	Sex	Primary / Secondary	Cytogenetics	IDH1	Other mutations
SU430	46	M	Primary	Der(7)t(7;11)	R132C	-
SU291.F	37	F	Primary	+8	R132H	FLT3-TKD, NPM1c+
SU277C	70	F	Primary	NK	R132G	
SU372	53	F	Primary	NK	R132H	FLT3-ITD
SU654	47	M	Primary	NK	R132C	NPM1c+
SU676	62	M	Primary	NK	R132H	NPM1c+, N-RAS, DNMT3A
SU582	71	F	Primary	NK	R132H	FLT3-ITD, N-RAS, NPM1c+
SU694	80	M	Secondary	+8	R132H	N-RAS
SU839	62	M	Primary	NK	R132H	FLT3-TKD
SU366	75	M	Secondary, MDS	NK	R132C	
SU223	18	F	Primary, Relapsed	t(9;11)	WT	MLL rearrangment
SU353	65	M	Primary	NK	WT	FLT3-ITD, NPM1c+
SU354	65	M	Primary, relapsed	+19	WT	
SU463	56	F	Primary	Complex	WT	K-RAS
SU674	51	F	Primary	NK	WT	DMNT3A R882H

d

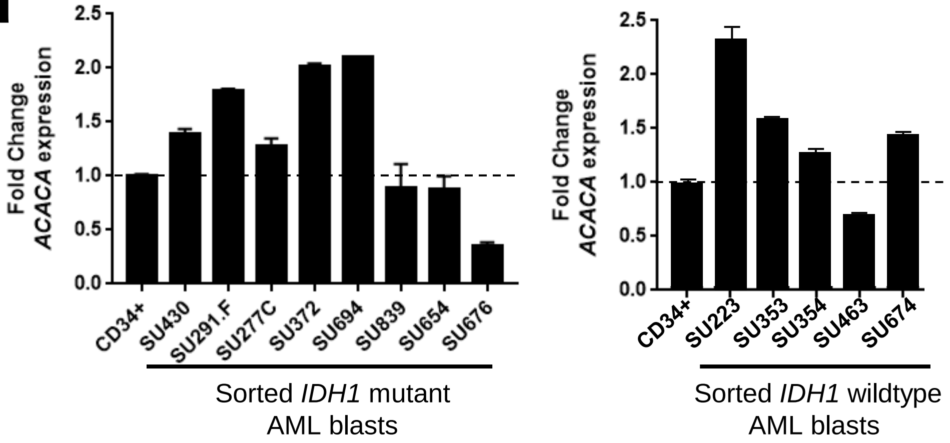

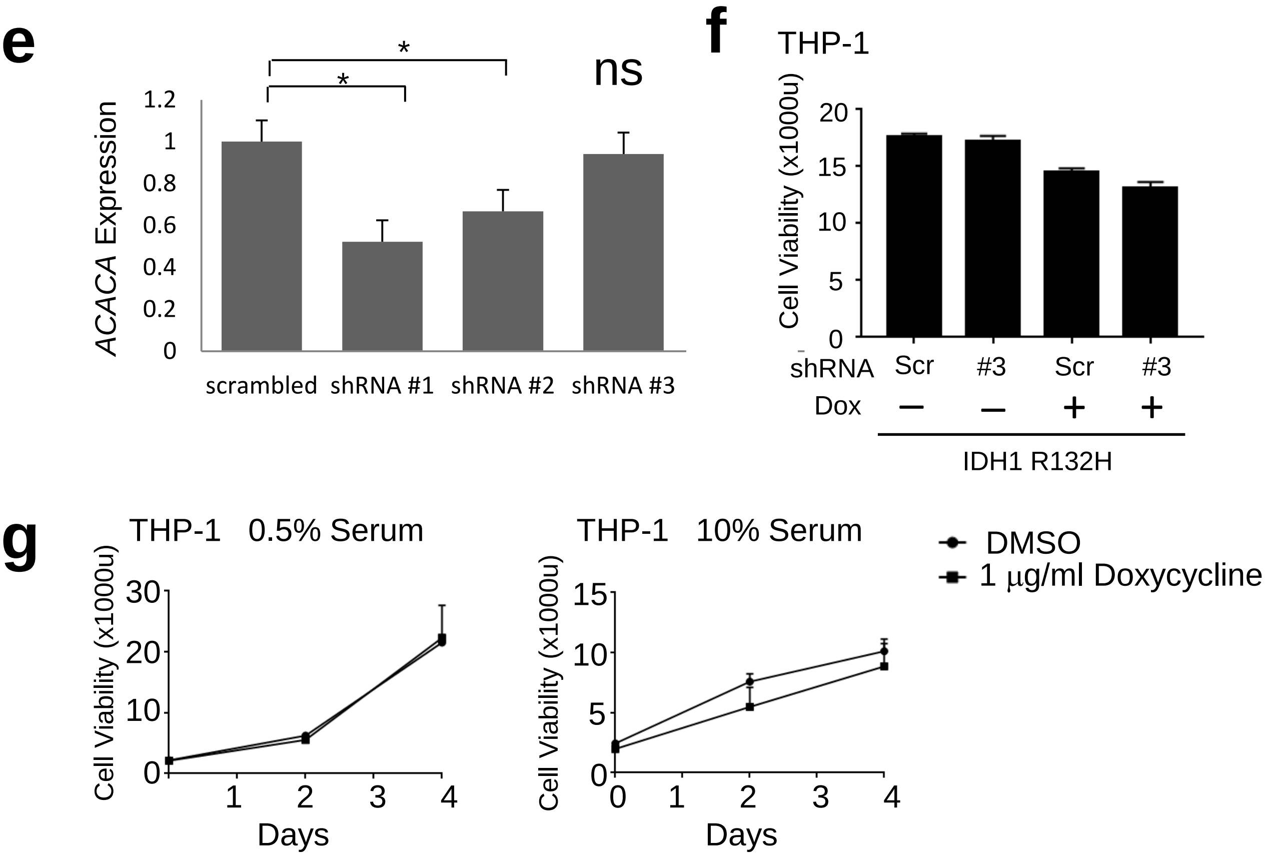
e
ACACA Expression
1.2
1
0.8
0.6
0.4
0.2
0
*
*
ns
scrambled
shRNA #1
shRNA #2
shRNA #3
f
THP-1
Cell Viability (x1000u)
20
15
10
5
0
shRNA
Scr
#3
Scr
#3
Dox
–
–
+
+
IDH1 R132H
g
THP-1 0.5% Serum
THP-1 10% Serum
DMSO
1 μg/ml Doxycycline
Cell Viability (x1000u)
30
20
10
0
15
10
5
0
1
2
3
4
Days

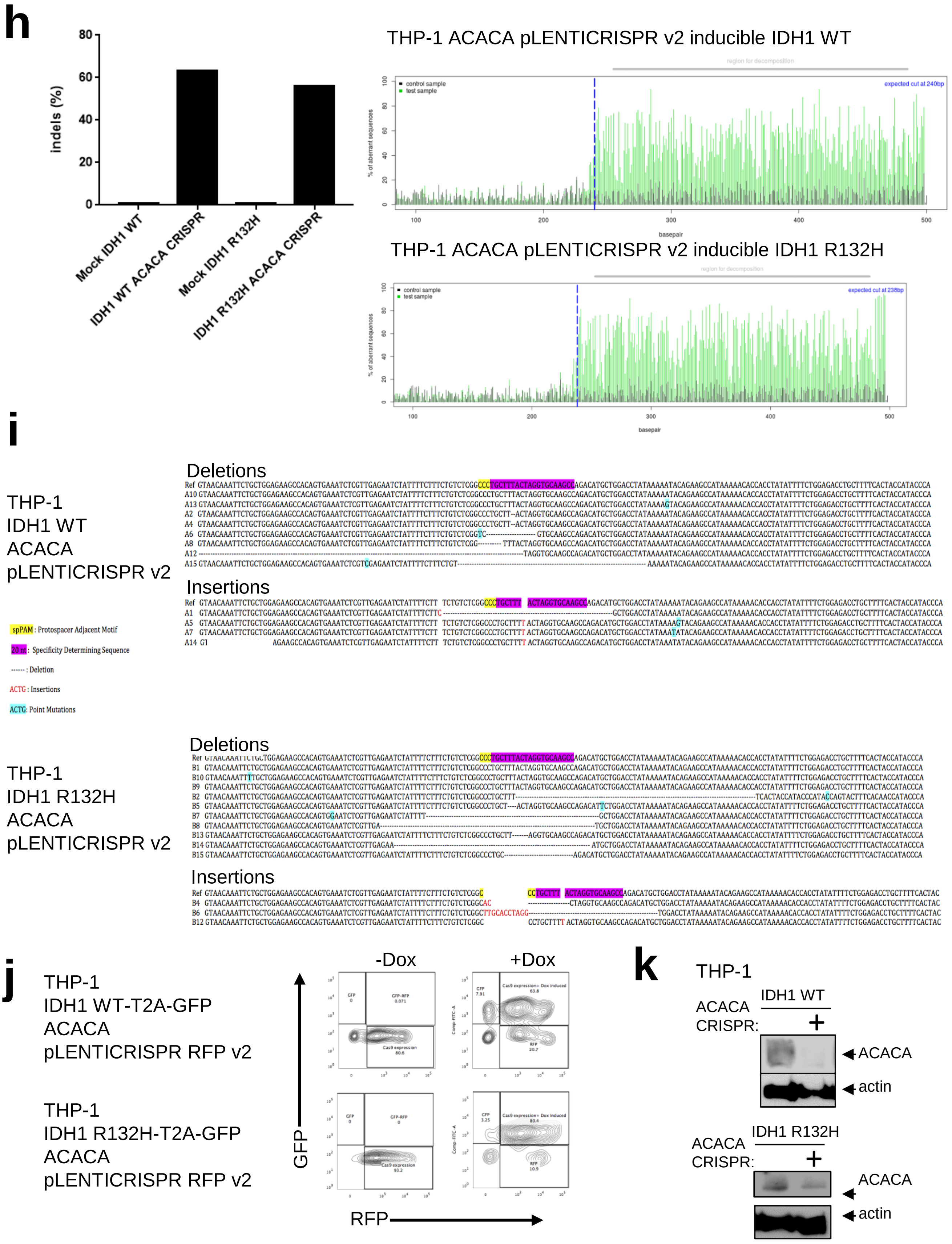
h
indels (%)
Mock IDH1 WT
IDH1 WT ACACA CRISPR
Mock IDH1 R132H
IDH1 R132H ACACA CRISPR
THP-1 ACACA pLENTICRISPR v2 inducible IDH1 WT
control sample
test sample
expected cut at 240bp
% of aberrant sequences
basepair
THP-1 ACACA pLENTICRISPR v2 inducible IDH1 R132H
expected cut at 238bp
i
THP-1
IDH1 WT
ACACA
pLENTICRISPR v2
Deletions
Insertions
spPAM : Protospacer Adjacent Motif
20 nt : Specificity Determining Sequence
------ : Deletion
ACTG : Insertions
ACTG: Point Mutations
THP-1
IDH1 R132H
ACACA
pLENTICRISPR v2
Deletions
Insertions
j
THP-1
IDH1 WT-T2A-GFP
ACACA
pLENTICRISPR RFP v2
THP-1
IDH1 R132H-T2A-GFP
ACACA
pLENTICRISPR RFP v2
-Dox
+Dox
GFP
RFP
k
THP-1
IDH1 WT
ACACA
CRISPR:
+
ACACA
actin
IDH1 R132H
ACACA
CRISPR:
+
ACACA
actin

l

TOPO Sequencing Exon 4 *ACACA* THP-1 Clones

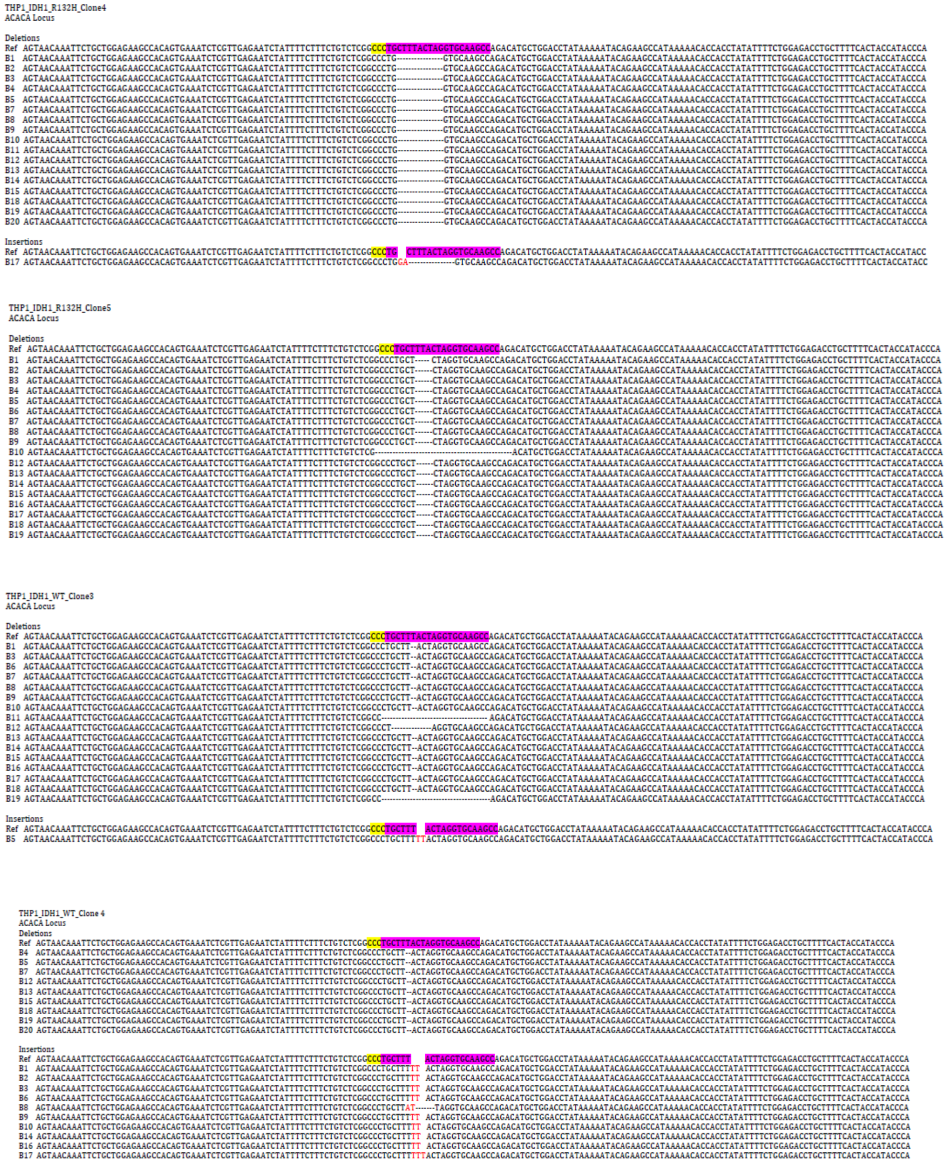

m

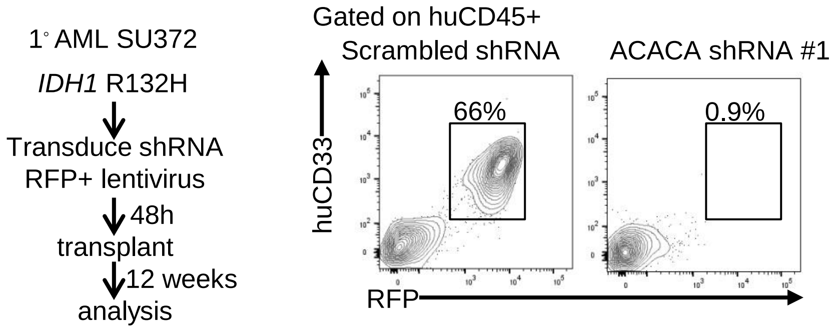

a

Panobinostat

HDAC1, HDAC2, HDAC3, HDAC4, HDAC5, HDAC6, HDAC7, HDAC8, HDAC9, HDAC10, HDAC11, SIRT1, SIRT2, SIRT3, SIRT4, SIRT5, SIRT6, SIRT7

AZD6244, PD-0325901

MAP2K1, MAP2K2, MAP2K3, MAP2K4, MAP2K5, MAP2K6, MAP2K7

b

Target	Drug	Evaluable	p-value
HDAC	Panobinostat	Yes	0.004
MEK	AZD6244, PD-0325901	Yes	0.01
RTK	Sorafenib	Yes	0.09
CDK4	PD-0332991	No, very few sensitive lines	-
RAF	PLX4720, RAF265	Yes	NS (> 0.1)
EGFR	Erlotinib, Lapatinib, ZD-6474	Yes	NS (> 0.1)

c

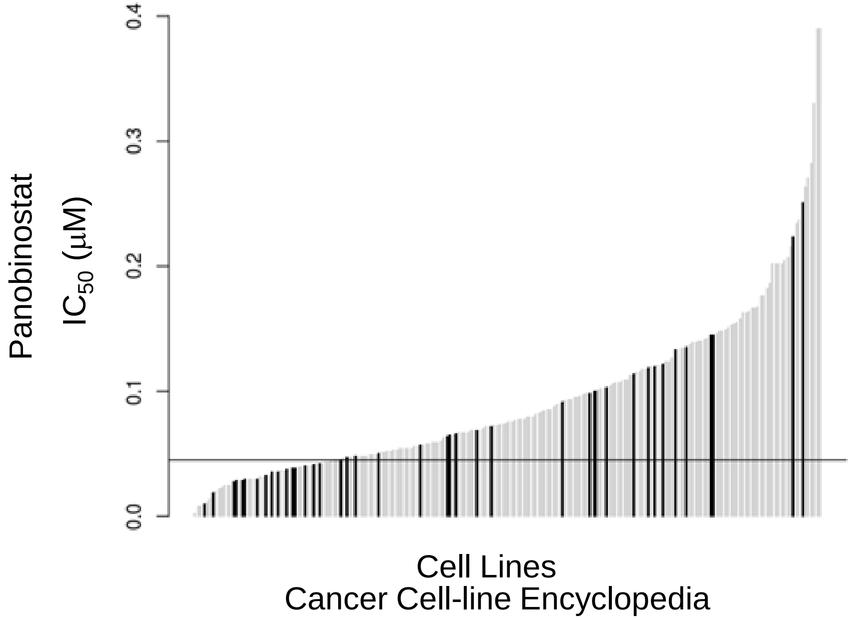

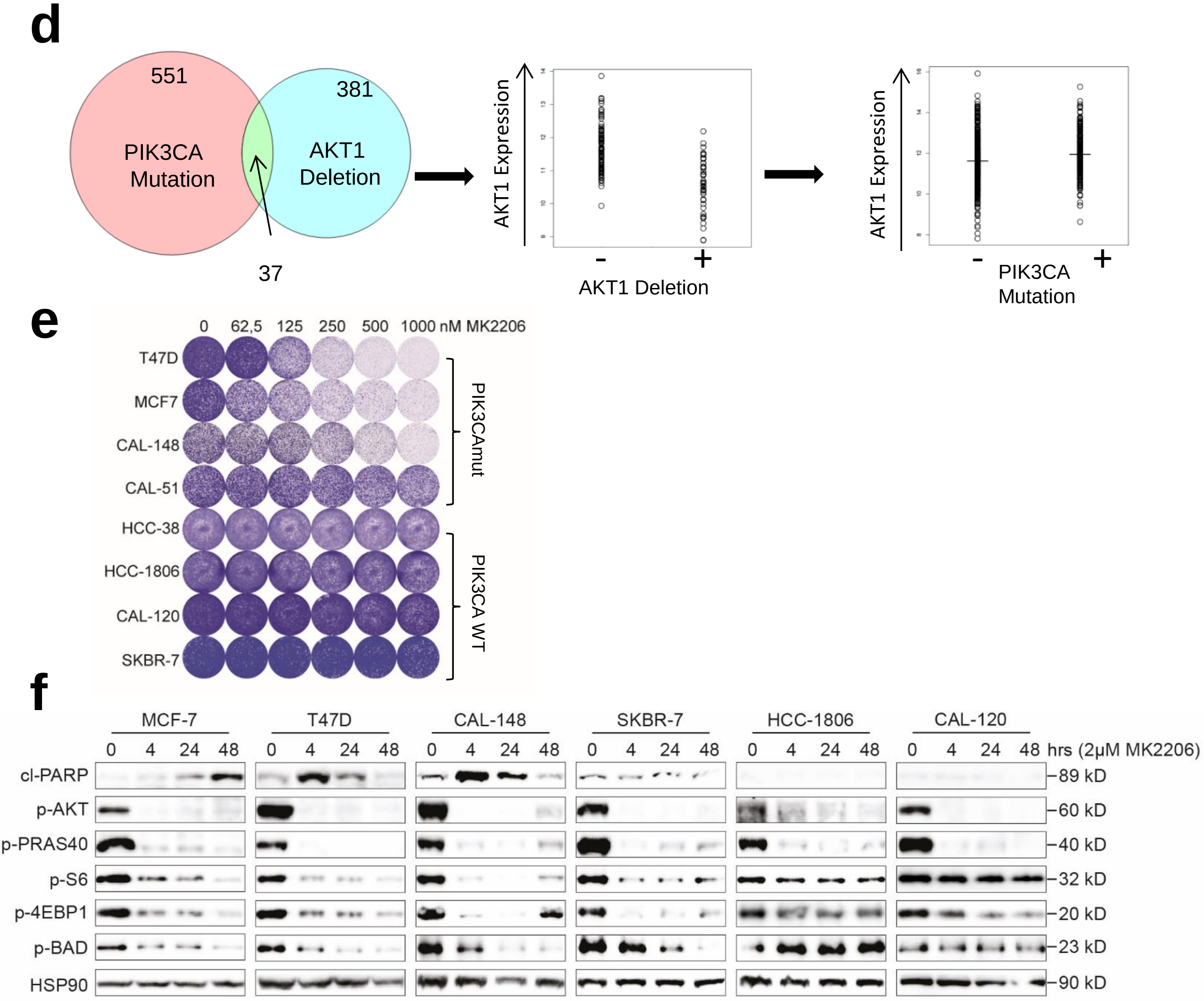

Supplementary Figure 5. Validation of Predictive Biomarker Analysis using MiSL. (a) Targets of Panobinostat (HDAC inhibitor) (upper panel) and MEK inhibitors AZD6244 and PD-0325901 (lower panel) as per DGidb data. (b) Summary of comparisons between MiSL-predicted sensitive cell lines and sensitive cell lines using pharmacologic data for a variety of targets. There is a statistically significant overlap between the predictions and sensitivity based on actual pharmacological data. (c) Plot showing IC_{50} values for all the CCLE cell lines (ranked in decreasing order of sensitivity) tested by Barrentina et. al.. for Panobinostat (HDAC inhibitor). The horizontal line shows the IC_{50} threshold used to determine sensitivity to the drug – lower than threshold implies the cell line is sensitive. The MiSL-predicted sensitive cell lines are marked in black. (d) MiSL analysis steps illustrated for *AKT1* – (i) mutual exclusion of *PIK3CA* mutation and gene deletion across cancers (HI-LO Boolean implication), (ii) deletion of gene concordant with lower expression of gene ($p < 0.05$), (iii) expression of gene is higher in *PIK3CA*-mutated breast cancer ($p < 0.05$) (e) Breast cancer cells were seeded in the presence of increasing concentrations of MK-2206 with concentrations as indicated. All cells were fixed with 4% formaldehyde and stained with 0.1% crystal violet when wells containing untreated cells became confluent. (f) Western blot of cell extracts of *PIK3CA* mutant (MCF-7, T47D, CAL-148) and *PIK3CA* wild-type (SKBR-7, HCC-1806, CAL-120) breast cancer cells treated with 2 mM MK-2206 for 0, 4, 24 and 48 hours blotted with antibodies against cleaved-PARP, phospho-AKT, phospho-PRAS40, phospho-S6 kinase, phospho-4EBP1, phospho-BAD and HSP90.

a

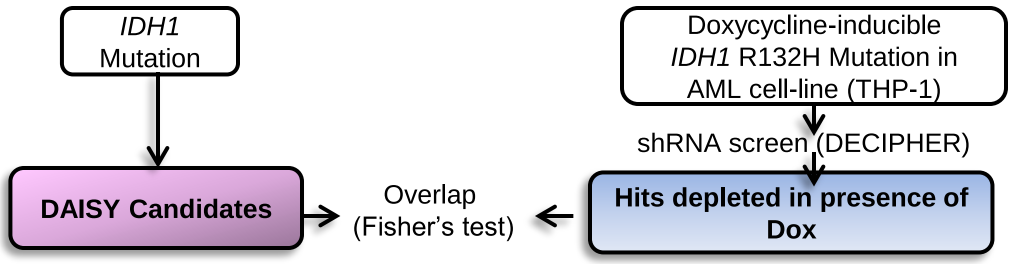

b

***IDH1* R132 mutation, AML**

	shRNA Hits	shRNA Non-SL
DAISY Candidates	27	203
Non-candidates	749	7200

p-value = 0.14

c

***IDH1* R132 mutation, AML**

	shRNA Hits	shRNA Non-SL
DAISY Candidates	12	218
Non-candidates	427	7522

p-value = 0.58

Supplementary Figure 6. DAISY Comparison with shRNA Data. (a) Schematic for comparing DAISY candidates for *IDH1* mutation with synthetic lethal partners as per DECIPHER library screen generated using a doxycycline-inducible IDH1 R132H THP-1 cells. (b) Contingency table showing overlap between DAISY candidates for *IDH1* mutation and shRNA synthetic lethals as per DECIPHER screen with drop-out ratio of 0.8 for 2 or more shRNAs for calling synthetic lethals in shRNA data. The overlap is not statistically significant. (c) Contingency table showing overlap between DAISY candidates for *IDH1* mutation and shRNA synthetic lethals as per DECIPHER screen with a more stringent criterion (drop-out ratio of 0.6 for 2 or more shRNAs) for calling synthetic lethals in shRNA data. The overlap is still not statistically significant with the more stringent criterion for calling synthetic lethals.

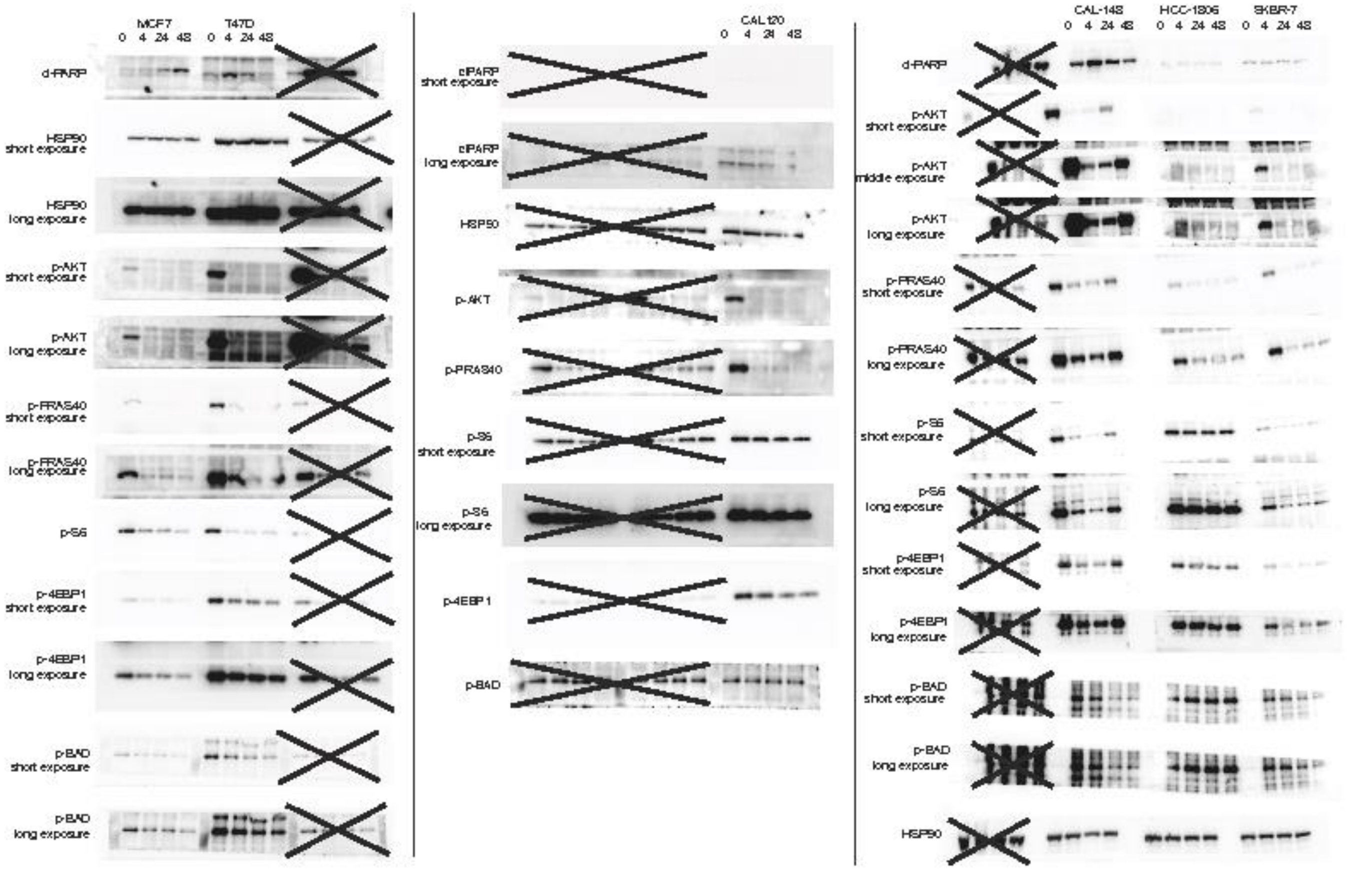

Supplementary Figure 7 Uncropped images of Western blot of cell extracts of *PIK3CA* mutant (MCF-7, T47D, CAL-148) and *PIK3CA* wild-type (SKBR-7, HCC-1806, CAL-120) breast cancer cells treated with 2 mM MK-2206 for 0, 4, 24 and 48 hours blotted with antibodies against cleaved-PARP, phospho-AKT, phospho-PRAS40, phospho-S6 kinase, phospho-4EBP1, phospho-BAD and HSP90.
